# Succinate Dehydrogenase is the Regulator of Respiration in *Mycobacterium tuberculosis*

**DOI:** 10.1371/journal.ppat.1004510

**Published:** 2014-11-20

**Authors:** Travis Hartman, Brian Weinrick, Catherine Vilchèze, Michael Berney, Joanne Tufariello, Gregory M. Cook, William R. Jacobs

**Affiliations:** 1 Department of Microbiology and Immunology, Howard Hughes Medical Institute, Albert Einstein College of Medicine, Bronx, New York, United States of America; 2 Department of Microbiology and Immunology, Otago School of Medical Sciences, University of Otago, Dunedin, New Zealand; McGill University, Canada

## Abstract

In chronic infection, *Mycobacterium tuberculosis* bacilli are thought to enter a metabolic program that provides sufficient energy for maintenance of the protonmotive force, but is insufficient to meet the demands of cellular growth. We sought to understand this metabolic downshift genetically by targeting succinate dehydrogenase, the enzyme which couples the growth processes controlled by the TCA cycle with the energy production resulting from the electron transport chain. *M. tuberculosis* contains two operons which are predicted to encode succinate dehydrogenase enzymes (*sdh-1* and *sdh-2*); we found that deletion of Sdh1 contributes to an inability to survive long term stationary phase. Stable isotope labeling and mass spectrometry revealed that Sdh1 functions as a succinate dehydrogenase during aerobic growth, and that Sdh2 is dispensable for this catalysis, but partially overlapping activities ensure that the loss of one enzyme can incompletely compensate for loss of the other. Deletion of Sdh1 disturbs the rate of respiration via the mycobacterial electron transport chain, resulting in an increased proportion of reduced electron carrier (menaquinol) which leads to increased oxygen consumption. The loss of respiratory control leads to an inability to recover from stationary phase. We propose a model in which succinate dehydrogenase is a governor of cellular respiration in the adaptation to low oxygen environments.

## Author Summary

This work establishes the principle that *Mycobacterium tuberculosis* undergoes a metabolic remodeling as oxygen concentrations fall that serves to decrease its rate of oxygen consumption and therefore oxidative phosphorylation. Furthermore, cells can be stimulated to respire, even in low oxygen conditions, by providing reducing equivalents to the respiratory chain by either genetic manipulation (deletion of succinate dehydrogenase) or by exogenous addition of reducing agents such as DTT. Thus, activation of persister cells may be accomplished by increasing their respiration rate in low oxygen conditions. These findings will inform the design of novel drug screens which should seek enhancers of cellular respiration to find compounds which will serve to shorten the duration of TB chemotherapy.

## Introduction

The World Health Organization has estimated the prevalence of Tuberculosis (TB) in the human population to be nearly two billion people. Although only a fraction of those individuals will ever display symptoms, TB is still a significant cause of worldwide mortality and was responsible for 1.3 million deaths in 2012 [1]. The organism responsible for this disease, *Mycobacterium tuberculosis*, owes its unqualified success as a pathogen to the ability to survive and persist in a human host, where it can evade immune surveillance and establish a sub-clinical infection. These latently infecting bacilli have the potential for reactivation in certain circumstances, as is commonly seen in HIV-induced immunosuppression [2]. In addition to immune evasion mechanisms found in some other chronic pathogens, *M. tuberculosis* appears to evade immunity by adopting a metabolically active but quiescent state during which cell division is limited [3]. In fact, the current antibiotic therapy regimen recommended by the WHO is multiphasic and is modeled around the presence of tolerant persister cells that are not cleared in the initial two months of treatment. The reliable occurrence of this subpopulation in clinical investigations has led to the addition of a continuation phase to the antibiotic course, which can last four months or more. Currently, the physiological adaptation which enables this organism to persist remains an area of active research, but targeting persisters should considerably improve the outcome of therapeutic efforts.

The inability to physically isolate a persister subpopulation without perturbing its labile state has prompted the adoption of a number of approaches to gain insight into the basis of the phenomenon. These models, which recapitulate a slowly- or non-dividing state in vitro, have revealed a number of interesting clues to persister physiology. It is important to note that *M. tuberculosis* is widely considered to be an obligate aerobe with the important stipulation that even though division is limited in anaerobic conditions, bacterial cultures can remain viable for decades [4]. A model developed by Wayne was instrumental in delineating the oxygen set points which result in cessation of division below 1% dissolved oxygen (DO), and a decline in survival below 0.06% DO, thus providing a framework to understand dormancy [5]. More recently, Gengenbacher et al. found that when quiescence is initiated through nutrient starvation the bioenergetic remodeling results in a decrease of ATP to one-fifth its log phase level, a concentration which apparently is reflective of maintenance of the protonmotive force [6]. Watanabe et al. subsequently verified these results and further noted that the depletion of ATP correlated with an apparent dearth of NAD^+^, at very low dilution rates in continuous culture [7]. The information gleaned from these studies directly informs the mechanistic descriptions of new TB drugs, including the diarylquinolone, Bedaquiline, a newly approved ATP-synthase inhibitor which is effective against dormant mycobacteria [8].

Although a number of studies have examined the transcriptional response of dormant cells, direct genetic evidence of metabolic genes essential for growth rate transitions was reported from studies of the abundance of specific mutants in transposon insertion libraries following alteration of the dilution rate in continuous culture. Among enzymes with a bioenergetic function, genes involved in energy metabolism (a putative succinate dehydrogenase), and a number of oxidoreductases were found to be important for this transition suggesting that the resumption of growth requires the benefits of oxidative phosphorylation [9]. It is difficult to point to a specific physiological adaptation which would be responsible for survival without knowledge of which of the diverse *in vivo* microenvironments might harbor persistent mycobacteria, but some groups have tried screening approaches aimed at addressing these questions in specific tissues [10], [11]. In terms of bioenergetic capacity, these studies revealed that one member of an operon containing a putative succinate dehydrogenase appeared to be essential for *in vivo* mycobacterial survival in a mouse model during the chronic phase of infection, a finding that was subsequently repeated using an analogous transposon-based screen [10], [12].

**Figure 1:**
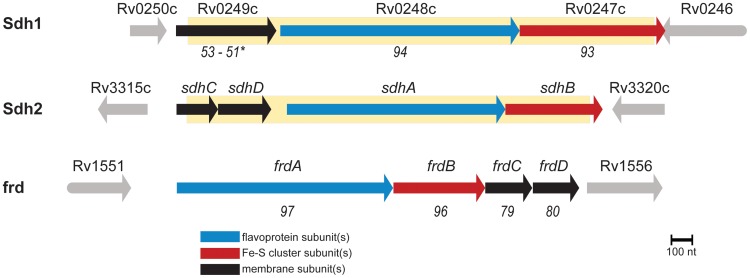
*M. tuberculosis contains three* enzymes capable of succinate oxidation. Homology scores are from pairwise alignments and reported in T-Coffee units (see methods) with respect to sdh2. (*) indicates homology scores of the fused membrane subunits of sdh1 with respect to sdh2 by pairwise alignment of *sdhC* and *sdhD*. Shaded yellow area indicates deleted portions of ORFs described in this work.

Oxidative flux through the TCA cycle is directly coupled to the electron transport chain via the oxidation of succinate and the corresponding reduction of membrane-localized quinones. Disruption of this activity would be a good strategy for control of growth in the energy limiting conditions that *M. tuberculosis* is thought to encounter *in vivo* [13]. This is an important consideration because ATP generation by oxidative phosphorylation is energetically much more efficient than ATP generation by substrate-level phosphorylation. *M. tuberculosis* has two operons annotated as succinate: quinone oxidoreductase - as well as a putative quinol:fumarate oxidoreductase which should be capable of succinate oxidation (see Annotation in Text S1). To date, the functional activity of these complexes has not been investigated, so we sought to understand their role in the transition from aerobic growth to persistence in *M. tuberculosis*. To this end, we targeted the two operons with homology to succinate dehydrogenase, which are encoded by *Rv0247c-Rv0249c* and *Rv3316–Rv3319* (Figure 1) (or according to the convention of Berney *et*. *al*. as *sdh1* and *sdh2*, respectively) [14], for further study.

In this work, we employed a combination of genetic, physiological and biochemical approaches to dissect the roles of Sdh1 and Sdh2 in the metabolic shiftdown of *M. tuberculosis* during adaptation to hypoxia. We report that Sdh1 (and not Sdh2) is the primary aerobic succinate dehydrogenase of *M. tuberculosis*. Deletion of this enzyme resulted in a number of bioenergetic deficiencies such as a major deficit in viability during stationary phase or during the chronic phase of infection in C3HeB/FeJ mice. The cause of this energetic insolvency was a peculiar mismanagement of oxygen consumption due to an imbalance in the redox state of the menaquinone pool. The Δ*sdh1* mutant consumed oxygen with close to perfect uncoupled kinetics, whereas wild type (*wt*) *M. tuberculosis* enacted an oxygen conservation strategy. The respiratory rate was dependent on the redox state of the menaquinone pool and respiration could be stimulated in non-respiring cells by adding exogenous reductant.

## Results

### *M. tuberculosis* strains lacking Rv0247c-Rv0249c have a survival defect in stationary phase

**Figure 2:**
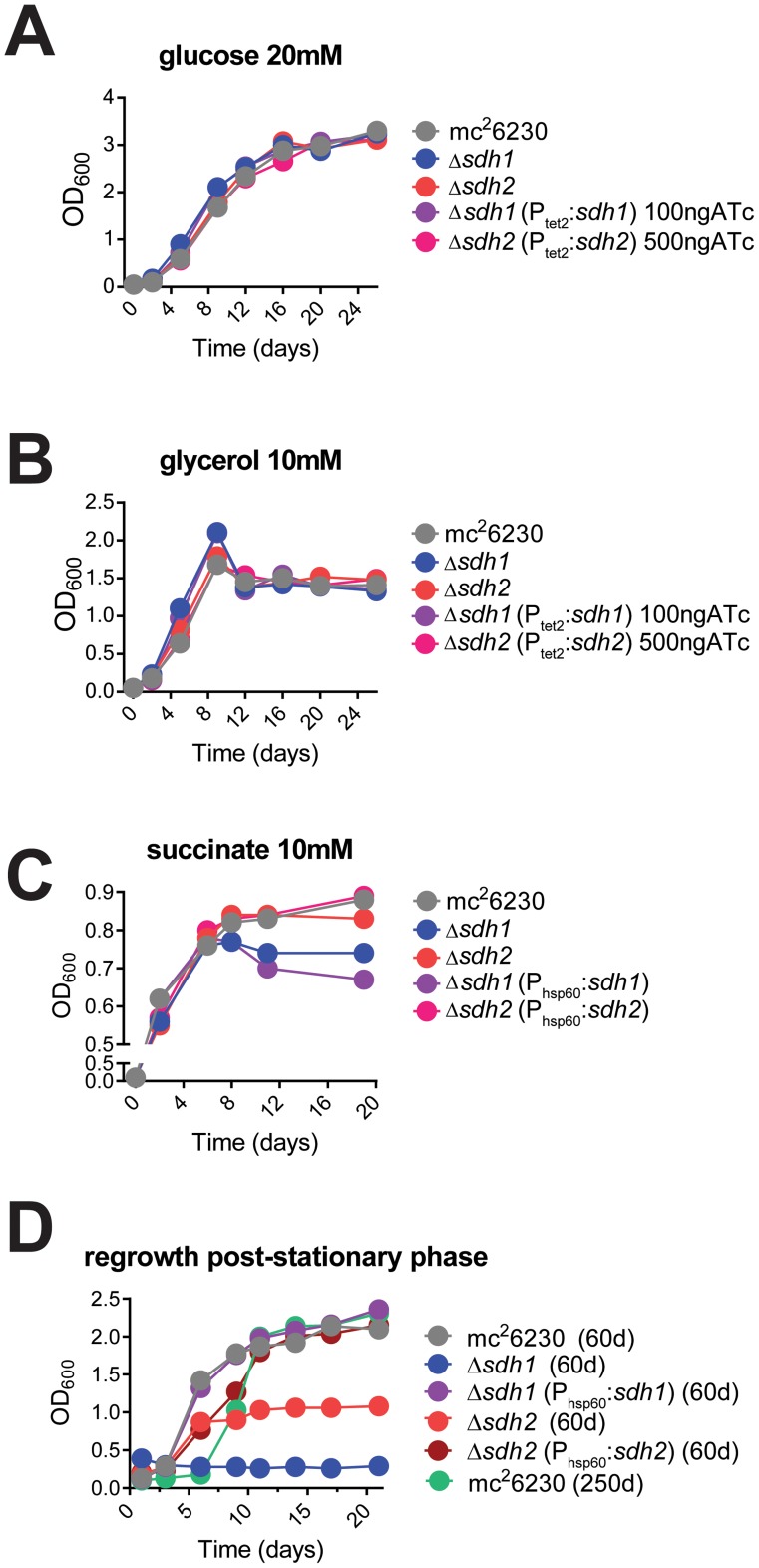
Logarithmic growth is unaffected in *sdh1* mutants, but cultures do not survive stationary phase. Growth of Δ*sdh* strains was monitored by recording OD_600_ periodically in cells grown in 7H9 media with glucose (A), glycerol (B), or succinate (C) as primary carbon sources. Δ*sdh* strains are able to grow with normal log phase kinetics in complete media (see Methods), but display a stationary phase exit defect after ∼60 days in culture (D). *M. tuberculosis* strains mc^2^6230 (parent), mc^2^7296 (Δ*sdh1*), mc^2^7297 (Δ*sdh2*), mc^2^7298 (Δ*sdh1*, P_hsp60::_ *sdh1*), or parental strain after>8 months of stationary phase were inoculated at OD_600_ 0.01 and grown to stationary phase in 7H9 complete media (see Methods) for the durations listed in the graph title, then subcultured to OD_600_ 0.1 in the same media. Single replicates are depicted that are representative of at least two independent experiments.

To determine the role of each enzyme complex, we prepared strains with null deletions of each in attenuated (mc^2^6230) and virulent (H37Rv) strains of *M. tuberculosis* using specialized transduction (Table S1 and Complementation in Text S1) [15], [16]. For safety reasons, we relied on null mutants of attenuated strains for all assays in which virulence was not a primary focus. The resulting mutant strains displayed no observable differences in growth rate in media containing glucose or glycerol as a primary carbon source (Figure 2A, B), however we observed a growth defect for Δ*sdh1* when succinate was the sole available carbon source compared to the parent or Δ*sdh2* (Figure 2C). These results were consistent for virulent and attenuated strains. In addition, we observed a stationary phase exit defect in which Δ*sdh1* was unable to be rescued from two-month old cultures, and the *sdh2* mutant grew poorly after a similar period (Figure 2D). The parental cultures or complemented strains exhibited no comparable decrease in growth rate or saturation even after eight months of stationary phase, indicating that these operons do not have perfectly redundant catalytic activities *in vitro*.

### The putative operon Rv0247c-Rv0249c encodes a succinate dehydrogenase

Succinate dehydrogenase catalyzes the two-electron oxidation of succinate to fumarate with a corresponding reduction of quinone to quinol, but physiologically, the succinate oxidation:fumarate reduction catalytic ratios are dependent on substrate concentrations and are critically dependent on the redox potential [17], [18]. Absolute pool sizes of metabolic intermediates are highly dynamic in living cells as a function of growth stage, pH, gas mixture, and temperature. As a result, the predominant direction of catalysis for each enzyme at any time cannot be inferred by annotation alone. In fact, the SDH reaction in mycobacteria should have an unfavorable free energy because the redox potential of menaquinone is lower than that of the succinate to fumarate reaction [19].

**Figure 3:**
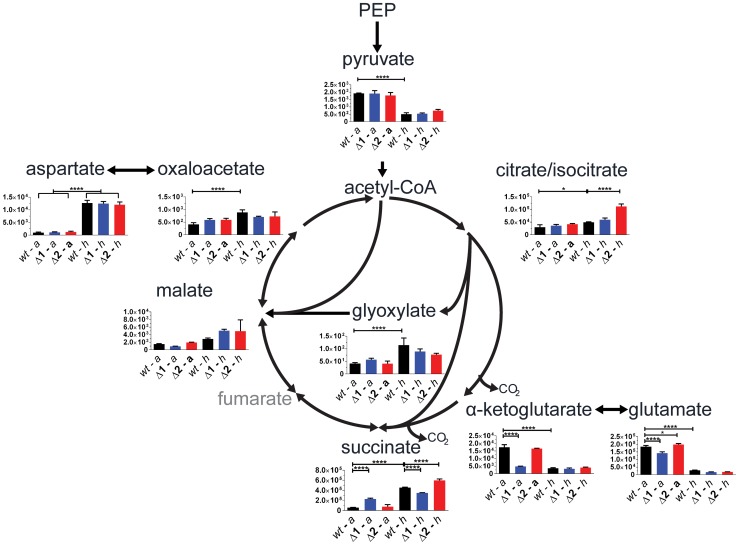
*sdh1* mutants prematurely accumulate succinate. For quantitation of intracellular metabolites from *M. tuberculosis*, parental or mutant cultures were grown to OD_600_ 0.5 (aerobic) and metabolites were extracted (see Methods in Text S1); or cultures were shifted into a hypoxic chamber for 12 days and metabolites were extracted without removal from the chamber. Data is reported as mean peak intensity ±SEM for each metabolite adjusted to OD_600_ for three replicate experiments from parental strain (*wt*), Δ*sdh1* (Δ*1*), or Δ*sdh2* (Δ*2*) strains in aerobic (*a*) or hypoxic (*h*) culture. Statistical significance determined by ANOVA, *, p<0.05; ****, p<0.0001. PEP, phosphenol pyruvate.

We evaluated gene function of the two *sdh* operons in a physiologically relevant context using a targeted metabolomic approach by analyzing differences in pool sizes of central carbon metabolites for cells in aerobic growth and in an anaerobic model [20]. Comparison of the mutant strains to the parental strain during aerobic growth revealed a significant 4-fold increase in intracellular succinate in Δ*sdh1* but no difference in Δ*sdh2*. This was accompanied by a 0.5-fold decrease of malate concentration in Δ*sdh1* compared to the parental strain, whereas the Δ*sdh2* strain showed no difference; these data suggest a loss of succinate dehydrogenase activity in the Δ*sdh1* strain (Figure 3). Consistent with observations made by others [21], we detected an accumulation in the total intracellular succinate concentration of the parental strain of *M. tuberculosis* of 8-fold after 10 days of anaerobiosis, while the concentration in Δ*sdh1* increases only 1.5-fold during this span. Conversely, total malate concentration rises slightly in the *wt* strain (1.7-fold), while the Δ*sdh1* mutant shows a 7-fold increase. The accumulation of intracellular succinate is suggestive of an inability of this strain to perform succinate oxidation, but since total concentrations of α-ketoglutarate decrease, and glyoxylate, oxaloacetate and malate increase in hypoxia, a portion of this succinate is likely to be from the reported activity of isocitrate lyase [21]. Consistent with this, during hypoxia we observed significantly less accumulated succinate in the Δ*sdh1* mutant relative to the parent (whereas Δ*sdh2* had an intracellular succinate concentration higher than the parent) and malate concentrations were 2.2 (for Δ*sdh1*) and 1.8-fold (for Δ*sdh2*) increased, though these differences were not significant.

We next verified that the aerobic accumulation of succinate in the attenuated *M. tuberculosis* mutants was reflective of the condition in the virulent strain using the same method. During aerobic batch culture, H37RvΔ*sdh1* and H37RvΔ*sdh2* accumulated succinate in excess of the parental H37Rv strain, and this accumulation was corrected for in the complementing strain (mc^2^7292) which constitutively expresses *sdh1*. This behavior is consistent with the complementation in the attenuated strains (see Figure S1, and Complementation in Text S1).

Based on these differences in metabolite pools, we analyzed the predominant direction of catalysis in the same aerobic and anaerobic models using stable isotope labeling (see Metabolomics in Text S1). Cells were grown in 7H9 medium supplemented with 10% OADC and labeled with [1,4-^13^C] aspartate in both four days of aerobic log phase growth and after twelve days in hypoxia using methods similar to those previously described [7]. We traced the fate of isotopically labeled carbon in TCA intermediates during aerobic growth (Figure S2A) and in hypoxia (Figure S2B) and determined the proportion of each labeled metabolite with respect to all isotopologues for each intermediate. The stable isotope labeling supported the classification of Sdh1 as an aerobic succinate dehydrogenase, but little difference in metabolite ratios was observed in strains lacking Sdh2 in these conditions.

We conclude from the metabolomic data that a functional reassignment should be considered for the operon encoded by *Rv0247c-Rv0249c*. We propose that *Rv0247c-Rv0249c* (Sdh1) encodes the primary succinate dehydrogenase of *M. tuberculosis* and the operon encoded by *sdhCDAB* (Sdh2) performs catalysis in an as yet undefined condition. To seek further support of this proposed classification, we analyzed gene expression of mutant strains in aerobic and hypoxic conditions (Table S2, and Methods in Text S1). Although no significant upregulation by the opposing *sdh* gene cluster was observed during aerobic growth, *sdh1* is significantly upregulated in Δ*sdh2* during anaerobiosis. Genes in the *sdh2* operon were not upregulated in Δ*sdh1* at either oxygen tension. This scheme is consistent with transcriptional data from oxygen-limited *M. smegmatis* that shows a 2-fold increase in *sdh2* transcripts but a 30-fold decrease of *sdh1* transcripts [14].

### Loss of SDH1 uncouples respiration and growth

**Figure 4:**
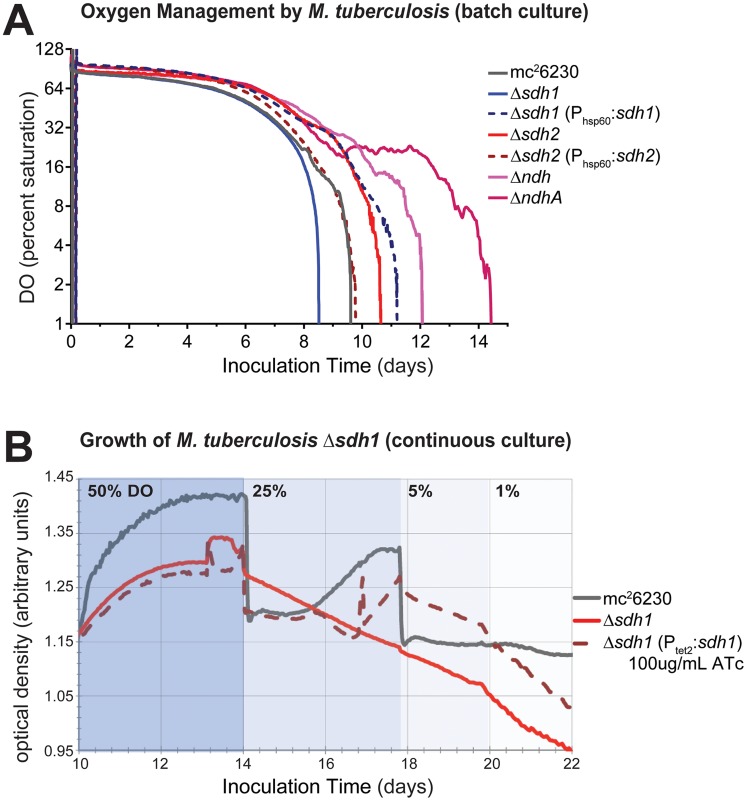
ETC mutants display disrupted oxygen consumption which negatively affects growth rates. (A) Respiratory kinetics were examined by monitoring dissolved oxygen (DO) consumption *of M. tuberculosis* strains in a bioreactor as available oxygen was consumed (see Methods). DO was monitored by oxygen probe after 24 h calibration in media alone and reported as percentage on log_2_ scale. (B) When *M. tuberculosis* strains are grown in continuous culture with 24 h^−1^ doubling times, washout of Δ*sdh1* occurs as DO concentrations are adjusted below 50%. Batch culture results are representative of four experiments.

As preservation of a proton motive force (PMF) is an important component of anaerobic survival, we monitored CFUs of *sdh* mutant strains in aerobic and anaerobic conditions in the presence of sub-lethal concentrations of the protononophore carbonyl cyanide m-chlorophenyl hydrazone (CCCP). Whereas 10 µM CCCP had a bacteriostatic effect on normoxic cultures (Figure S3A), the same concentration of CCCP resulted in a loss of viability of greater than 3-logs at 35 days of treatment (Figure S3B). Both mutant strains were more susceptible to PMF inhibition than the parental strain, but we were unable to recover colonies from Δ*sdh2* cultures after 21 days. This data supports the conclusion that Sdh2 is the generator of the PMF in hypoxia, as we have previously observed in *M. smegmatis* [22]. Next, we assessed the contribution of each *sdh* mutant to aerobic respiration in bioreactors operating in batch mode and in continuous culture. Cells were inoculated into a bioreactor system in which DO concentration, optical density, midpoint redox potential and pH could be measured simultaneously and were monitored throughout the growth curve as oxygen was depleted by the organism. Surprisingly, the parental strain initiated down-modulation of its respiration rate at ∼40% DO, while *Δsdh1* continued to respire unabated until the DO was entirely depleted (Figure 4A). Conversely, cells harboring a deletion of *sdh2* consumed oxygen at a reduced rate and were able to modulate respiration as DO was depleted to ∼6%. These experiments revealed that constitutive overexpression of the complemented strain using the *hsp60* promoter overcompensated the respiratory phenotype. In subsequent experiments, we found that the oxygen consumption curve could be complemented in a strain expressing *sdh1* with a novel integrated tet-responsive promoter (P_tet2_); at low levels of anhydrotetracycline inducer (25 ng/mL – see Figure S4A, Complementation in Text S1). We concluded that these induction levels reflect the concentration of active enzyme in each condition; therefore, perfect complementation would require levels of expression which closely match *wt* levels throughout the growth curve.

The increased oxygen consumption of Δ*sdh1* should result in an increased membrane potential and an increased growth rate. However, data collected during batch culture experiments revealed that the initial growth rate for Δ*sdh1* is actually slightly slower in aerobic conditions (Table 1); this rate decreases considerably once oxygen is depleted, yet the parental strain maintains faster population doubling times than either *sdh* mutant (Table 1, Figure S5). This apparent uncoupling of respiration from growth was further analyzed in a separate chemostat experiment. When cultures were grown with a 24 hr doubling time, Δ*sdh1* (but not the parental strain) was unable to maintain the growth rate at DO levels of 25%, 5%, or 1%, and consequently washed out of continuous culture (Figure 4B). Because the membrane potential (ΔΨ) is the major component of the PMF at neutral pH values, we assessed the membrane potential by measuring uptake of the lipophilic cation tetraphenylphosphonium (TPP^+^) [23]. In aerobiosis the ΔΨ was comparable between the *wt* and Δ*sdh* mutants (53–66 mV) (Table 2). In hypoxia, the ΔΨ was considerably higher (30 vs. 18 mV) for the Δ*sdh1* mutant compared to the *wt* and Δ*sdh2* strains (Table 2).

**Table 1:** Doubling times (hours^−1^) of mc^2^6230 strains at 100%→1%DO (aerobic rate), and at 1%→0% (hypoxic rate) during growth of batch culture in 200 mL bioreactors.

strain	mc^2^6230	Δ*sdh1*	Δ*sdh2*	Δ*ndh*	Δ*ndhA*
aerobic rate	27.9	32.9	30.9	28.6	32.9
hypoxic rate	123.4	146.5	153.4	163.8	n/a

**Table 2:** Membrane potential in mV of *M. tuberculosis* strains at OD_600_ 0.5 (aerobiosis), and after 12 days in hypoxia was measured by uptake of [^3^H]TPP^+^. Data are averages of biological triplicates.

strain	mc^2^6230	Δ*sdh1*	Δ*sdh2*	Δ*sdh1*:: pYUB1757 25 ng/ml ATc	Δ*sdh2*:: pYUB1756 100 ng/ml ATc
aerobiosis	52.8	66.1	60.3	82.3	85.0
hypoxia	18.3	30.2	8.0	76.8	*undetected*

### Respiratory control of *M. tuberculosis* is governed by the redox state of the menaquinone pool

Mycobacteria use menaquinone as their main electron carrier in the electron transport chain. Coupling succinate oxidation (E°′ ∼ +30 mV) to menaquinone reduction (E°′ ∼ −80 mV at pH 7) is an energetic challenge, because this reaction is endergonic [24]. A model to explain this conundrum posits that reversed electron transport across the cytoplasmic membrane can provide the energy required to drive the oxidation of succinate using the PMF [25]. This suggests that the increased respiration rate in the Δ*sdh1* strain is due to the absence of reverse electron flow and consequently an altered redox state of the quinone pool. To confirm the hypothesis that the respiratory rate is a function of the redox balance of the menaquinone/menaquinol pool we sought corroborating evidence using *M. tuberculosis* strains harboring deletions of the type II NADH dehydrogenases *ndh* and *ndhA*. These enzymes are thought to be the primary means of electron input in *Mycobacteria* [26] during aerobic growth. A down-modulation of oxygen consumption by these strains occurred at ∼50% and ∼10%, respectively (see Figure 4A). Complementation of Δ*ndhA* was similar to that of the *sdh* enzymes with overcomplementation of oxygen consumption when *ndhA* was expressed episomally using a constitutive promoter (Figure S4B), further illustrating the necessity of “fine tuning” respiratory enzyme levels to achieve maximal growth. This finding supports the paradigm that enzyme activities that facilitate rapid reduction of the quinone pool serve to *increase* the respiratory rate in the *wt* strain (since their deletion reduces oxygen consumption), and fumarate reduction functions as a respiratory brake during aerobiosis by an opposing oxidation of the pool. The unexpected disparity in DO-sensitive modulation of respiration by the type II NADH dehydrogenases suggests a wider strategy to indirectly sense oxygen concentrations in the immediate environment and spend reducing equivalents accordingly before taking the drastic step of uncoupling biomass production from respiration.

**Figure 5:**
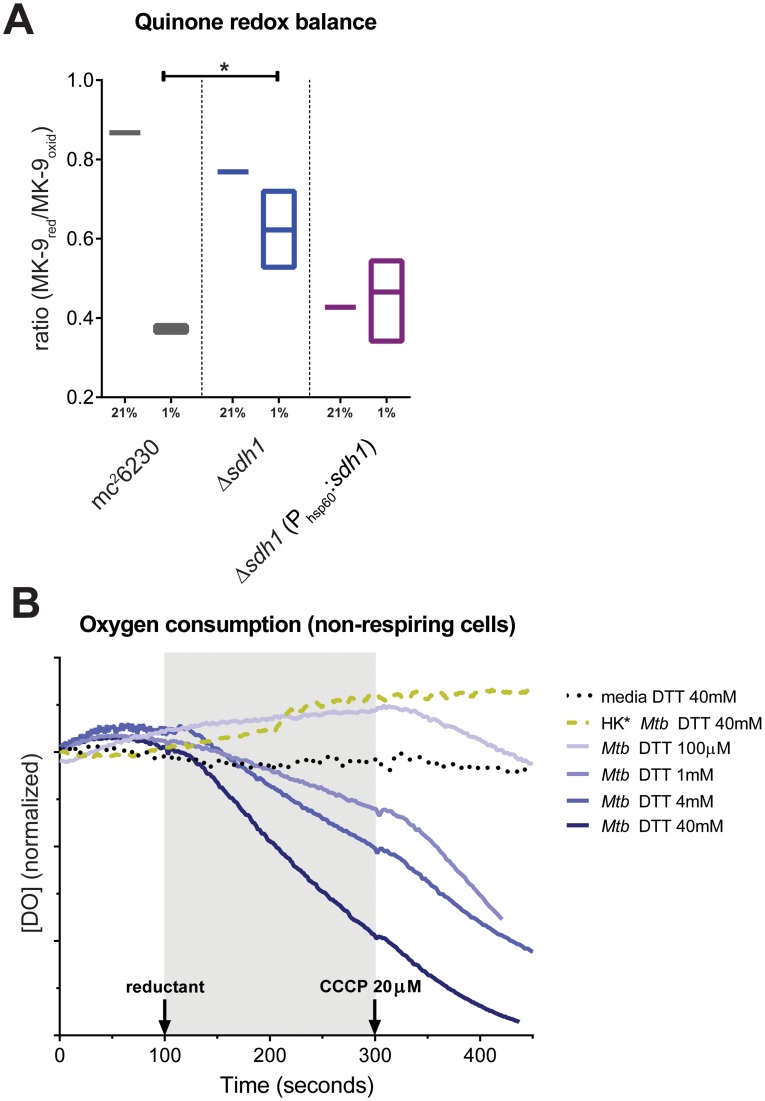
Δ*sdh1* maintains balanced quinone poise at low [O_2_], but can be stimulated to respire. Menaquinone redox poise (A) was measured by extraction of total quinone from cells grown in bioreactors operating in batch mode in which dissolved oxygen was maintained at ambient levels (21%) or at 1% (entry into NRP-1). Proportion of MKH_2_:MK is reported as mean ±SEM of three replicates. Addition of DTT stimulates oxygen consumption of non-respiring *M. tuberculosis* cells (B). To detect initiation of oxygen consumption by reductants, 5 mL cells were added to the incubation chamber of a Clark-Type oxygen electrode and basal oxygen consumption was monitored for 100 seconds, at which point compound was added. After 200 seconds, maximal uncoupled oxygen consumption rate was determined by the addition of 20 µM CCCP for 100 seconds (see Supplementary Methods). Dissolved oxygen concentration was normalized to the level determined at the start of the experiment; each tick on the y-axis represents 10% DO of air-saturated media. Media alone and heat killed (HK*) cells were used as controls.

The apparent diminution of succinate oxidation in Δ*sdh1* during aerobiosis, and its uncontrolled respiratory phenotype alluded to an imbalance in the redox state of the menaquinone pool. We sought to confirm this biochemically by extracting menaquinones (MK-9) from cells growing aerobically, and at 1% DO in bioreactors (see Methods). Ratios of menaquinol:menaquinone of the parental strain were balanced when grown aerobically, but heavily skewed toward the oxidized state at low DO, conversely Δ*sdh1* had higher concentrations of menaquinol (reduced form), which was sufficient to drive respiration even at low oxygen levels (Figure 5A). In aerobically growing cells, we found the quinone pool to be balanced (ratio_MK-9red/MK-9oxid_  = 0.87), indicating equilibrium between respiratory rate and carbon flux. Because the balance of quinone reduction can shift rapidly, we sought further confirmation by monitoring data from a probe for midpoint redox potential. Cultures were grown in a bioreactor running in batch mode as described above but flowed compressed air into the bioreactor at 1L/hr. Using this measure, *M. tuberculosis* can be seen to utilize available oxygen then switch off respiration until oxygen builds up to a threshold concentration before switching on aerobic respiration again. Importantly, increases in the redox potential precede the onset of oxygen consumption by several minutes during which pH does not change (Figure S6 and S7); supporting the hypothesis that oxygen consumption is managed by quinone redox balance. Δ*sdh2* behaves in a manner similar to *wt*, but Δ*sdh1* appears to maintain a negative midpoint redox potential and respires all available dissolved oxygen without allowing it to build up in the vessel (Figure S8).

The above behavior is consistent with previous reports that respiratory rate can be directly controlled with first-order kinetics by the degree of reduction of the quinone pool in membrane vesicles and mitochondria [27], [28]. We took advantage of the relatively low midpoint redox potential of menaquinone [29], and sought evidence that the respiratory rate of intact mycobacterial cells could be stimulated using the membrane permeable reducing agent dithiothreitol (DTT). We hypothesized that cells which have entered the phase of respiratory downshift brought on by low oxygen concentrations should be stimulated to respire if menaquinol can be replenished by an exogenously applied reducing agent. To test this, *M. tuberculosis* strain mc^2^6230 was grown to early stationary phase and oxygen consumption was monitored in a Clark-type oxygen electrode (see supplementary methods) with and without the addition of DTT (Figure 5B). Stimulation of oxygen consumption was observed up to concentrations of 40 mM reductant, after which little increase was observed. Importantly, no stimulation of oxygen consumption was noted in media alone or in preparations of heat-killed cells from the same culture. No synergistic increase in oxygen consumption was observed in log phase cells in similar experimental conditions when the starting DO of culture media was greater than 50% that of aerated media, i.e. cells that are already respiring at maximal rates are not induced to respire faster by the addition of reductant.

### Loss of succinate dehydrogenase activity leads to decreased virulence and a survival deficit in lungs of C3HeB/FeJ mice

**Figure 6:**
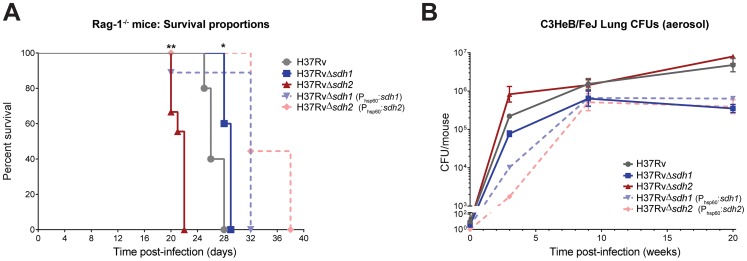
Modulation of succinate levels affects both virulence and survival of *M. tuberculosis* in mice. Virulence of mycobacterial strains was assessed by intravenous infection of five Rag-1^−/−^ mice (A) using H37Rv, H37RvΔ*sdh1* (mc^2^7292), H37RvΔ*sdh2* (mc^2^7293), H37RvΔ*sdh1*::*sdh1*(mc^2^7294), and H37RvΔ*sdh2*::*sdh2*(mc^2^7295) and survival was monitored over time. Data was analyzed by Gehan-Breslow-Wilcoxon test, where survival of mice infected with Δ*sdh1* (p = 0.0189) or Δ*sdh2* (p = 0.0011) and complementing strains were deemed significantly different with respect to H37Rv. Survival during chronic infection (B) was assessed by monitoring CFUs in the lungs of C3HeB/FeJ mice over time using the strains listed above. Data are means ± s.d. from four mice per time point per group.

To assess the effect of disregulated respiratory activity on pathogenesis and persistence, we tested the ability of *Mtb* Δ*sdh1* and Δ*sdh2* to cause disease in several established murine models. Previous experiments utilizing a high-throughput genetic screen have revealed subunits of *sdh1* (but not *sdh2*) to be underrepresented in the lungs of C57Bl/6J mice during chronic infection [10], [30]. It is not clear if the fitness defect observed in those screens is the result of reduced virulence or an inability of the mutants to maintain their numbers during chronic infection, but we were unable to recreate this phenotype with null deletion strains using the C57Bl/6J mice (Figure S9). To assess virulence, we infected immunodeficient Rag-1^−/−^ mice via low-dose aerosol; these mice produce no mature T or B cells and are thus unable to control mycobacterial infection [31]. Whereas immunodeficient mice infected with H37Rv had a median survival time of 26 days, Δ*sdh1* infected mice had a slightly longer median survival time of 29 days. Interestingly, the Δ*sdh2* strain displayed a hypervirulent phenotype; these mice had a median survival time of only 22 days (Figure 6A). The overexpressing complemented strains for both of these deletions were less lethal than either the mutants or the parental strain.

Given the predictive constraints of the mouse model in TB infection, particularly the inability of the murine immune system to form fibrous caseous granulomas [32], we think that any impact of these mutations on survival (or strains harboring deletions in respiratory enzymes) could be lessened because oxygen levels are likely always sufficient for growth in the murine lung. A murine model was developed to address this limitation; the C3HeB/FeJ mouse is an inbred strain that develops fibrous encapsulated lung lesions post-aerosol infection which appear to contain hypoxic centers [33]. We reasoned that the respiratory mismanagement of Δ*sdh1* would lead to a survival deficit in the lesions of mice containing hypoxic lesions. To test this hypothesis, we infected C3HeB/FeJ mice via aerosol and monitored burden over time (Figure 6B). By twenty weeks of infection, Δ*sdh1* had tenfold fewer cells per lung than the H37Rv parent (5.79 log_10_ CFU vs. 6.67 log_10_ CFU) and Δ*sdh2* was similar to the *wt*. It is important to note that after nine weeks the Δ*sdh1* burden dropped slightly, while *wt* cells continued dividing until week twenty. This suggests that deletion of Sdh1 leads to an inability to maintain bacterial numbers in the host, however, the difference in bacterial burden between *wt* and Δ*sdh1* was not as dramatic as we would have expected based on our *in vitro* results. This might be explained by the fact that gross pathology of upper lungs at twenty weeks did not reveal encapsulated granulomatous) lesions (Figure S10), thus oxygen was likely not restricted in the lungs of these mice.

## Discussion

The bioenergetic program that sustains *M. tuberculosis* during latency and in models that recapitulate persistence is of great interest because this survival is likely due to inhibition of growth that stems from an idle metabolic state [3], [34]. A mechanistic understanding of quiescence is of crucial importance to the planning of new antitubercular compound screens, which can be designed to directly target this population. To this end, we sought to understand the function of the enzyme responsible for the direct coupling of anabolism via the TCA cycle and the electron transport chain - succinate dehydrogenase. Prior to this work, the individual roles of the two predicted succinate dehydrogenases of *M. tuberculosis* had not yet been experimentally determined, and no obvious phenotype was reported in *M. tuberculosis* H37Rv containing a null deletion of the hypoxia-upregulated fumarate reductase, *frdABCD* [7]. Genetic manipulation of *M. tuberculosis* followed by an intracellular metabolomic approach allowed us to probe the functions of the two annotated Sdh enzymes and their role in cell physiology. Importantly, these enzymes were found to strongly influence aerobic respiration, and deletion of *sdh1* resulted in an increased rate of respiration, but did not result in faster cell growth. The work presented here validates the predicted role of *sdh1* as the primary succinate dehydrogenase during aerobiosis

It has been almost eighty years since Loebel and colleagues formally noted the capacity of *M. tuberculosis* for curtailing its oxygen consumption under anaerobic or starvation conditions [35], but a mechanism for this phenomenon is absent from the literature. Two distinct phases of adaptation to decreasing oxygen tension have been described; NRP (*non-replicating persistence*) stage 1 - marked by the cessation of cell division at ∼1% oxygen, and NRP stage 2 – a quiescent state occurring below 0.06% oxygen in which biomass production ceases [36]. Our data imply that *M. tuberculosis* employs an orchestrated respiratory slowdown as oxygen levels fall; this program is initiated while oxygen is still plentiful. The respiratory rate is fine-tuned by the opposing activity of the succinate dehydrogenase and fumarate reductase activities to maintain an optimal growth rate. This suggests that this tuning is controlled by balancing substrate concentrations, as has been suggested in electrochemical analysis of isolated enzymes [37], post-translationally [38], and via catabolite repression [39]. Management of respiration has important consequences for the proclivity for survival of *M. tuberculosis* amid a range of pathological niches in which oxygen tension can vary significantly, because ATP generation is much more efficient when electrons are committed to oxidative phosphorylation than through substrate-level phosphorylation alone.

**Figure 7:**
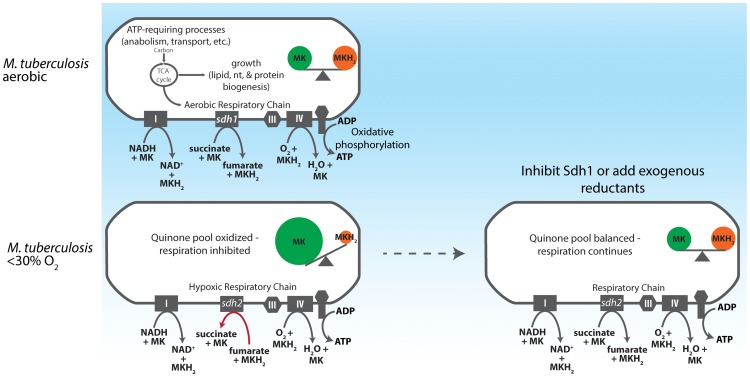
Model for redox control of respiration in *M. tuberculosis*. As an obligate aerobe, *M. tuberculosis* relies on oxidative phosphorylation to meet energy demands during growth phases. The organism accomplishes this by shuttling electrons into the ETC via the NADH dehydrogenases (Complex I) and succinate dehydrogenases. In aerobic conditions this electron flux both powers the electrochemical gradient and generates sufficient ATP to power cell division. As oxygen becomes restricted, Sdh1 (the primary aerobic succinate dehydrogenase) is inhibited and succinate accumulates intracellularly, thus depriving the ETC of a substantial source of electrons. Menaquinol is not replenished and oxygen reduction by the terminal cytochrome oxidases is diminished. Importantly, this process begins when DO concentrations drop below 30% - long before oxygen becomes limiting for growth. If Sdh1 is inhibited, intracellular succinate accumulates prematurely and the succinate oxidation must be catalyzed by Sdh2 or Frd. This results in unregulated menaquinone reduction which proceeds (even in oxygen replete conditions) to furnish the terminal oxidases with a substrate for oxygen reduction and respiration continues. (IV  =  Complex IV).

We favor a simple mechanistic explanation for the controlled respiratory slowdown that is consistent with structural studies of the terminal cytochrome *c* oxidase complex and the progression of the Q-cycle (Figure 7) [40]–[42]. Organisms will respire at optimal rates with a balanced quinone pool in which quinol (reduced) is present in sufficient concentration to immediately occupy the center P of the cytochrome oxidase complex; but when quinol is limiting - in an oxidatively skewed pool - respiration will progress at a less-than optimal rate. Figure 5A shows that whereas the *wt* strain has a largely oxidized quinone pool at 1% DO, the Δ*sdh1* mutant maintains a balanced pool, resulting in unchecked oxygen consumption. These data support a mechanism for respiratory downshift in *wt M. tuberculosis* that works as follows: as oxygen concentration drops below 40–30%, succinate oxidation also decreases leading to its buildup (hypoxic cells accumulate a sevenfold increase in intracellular concentration). This ‘unrespired’ succinate does not contribute to the reduction of membrane menaquinones, and as the ratio of menaquinol:menaquinone decreases from the activity of other electron donors, the cytochrome oxidoreductase is deprived of its substrate, thus decreasing the rate of oxygen consumption. However, the loss of the primary means of succinate oxidation in Δ*sdh1* results in a premature accumulation of succinate during aerobiosis that is partially relieved by Sdh2, which can function as a succinate dehydrogenase when cytosolic substrate concentrations favor this reaction. Published measurements of ubiquinone pools in *E. coli* show a similar trend [43] in quinone poise as oxygen decreases; but in that facultative anaerobe, reduced quinone increases as cells approach anoxia. The observation that a strong reductant can stimulate respiration of poorly respiring cells (Figure 5B) provides additional evidence of a menaquinol-limiting respiratory scheme. To our knowledge, this is the first time that any group has shown that oxygen consumption can be stimulated in living organisms that have shut off respiration by provision of exogenous electrons.

The oxygen consumption profiles of the two *sdh* mutants revealed another interesting aspect of mycobacterial physiology, a downshift of respiratory activity was initiated by the parental strain in the range of 40–30% DO. The decline in the rate of respiration indicates that the organism switches to a less thermodynamically efficient mechanism for ATP production as oxygen levels drop – but are still sufficient for growth. Current understanding of the mechanics of the decline in respiratory activity exhibited by *M. tuberculosis* upon adaptation to anaerobic conditions has been guided by analysis of the transcriptome of cells as they pass into hypoxia in various models [14], [44], [45]. Here we report that *M. tuberculosis* accomplishes gross control of aerobic respiration by depriving the cytochrome *c* oxidoreductase of menaquinol via a slow electron flux through Sdh1 and demonstrate how carbon passing through the TCA cycle is subject to this mechanism that couples growth and electron transport (Figure 7). Importantly, this modulation in the rate of oxygen consumption occurs long before oxygen becomes limiting for growth [26], and is absent any exogenously-provided inhibitor of respiration.

This respiratory management scheme should have direct *in vivo* relevance considering that physiological oxygen levels are only a fraction of those commonly used in *in vitro* culture models, and reflect the point at which we observe a downmodulation of oxygen consumption [46]. This might explain the pathological preference of *M. tuberculosis* for the upper lobes of the lung [47] where mycobacterial cellular respiration can function more efficiently. As cells are carried into tissues farther from the lung epithelia, oxygen becomes scarce and cells are forced into a less efficient bioenergetic program which could lead to decreasing ATP production and more reliance on glycolysis, β-oxidation, or storage compounds to meet energy demand. Aerosol infection of C3HeB/FeJ mice using the Δ*sdh1* strain led to a tenfold reduction in CFUs in the lungs (Figure 6B). Given the limitations of the murine TB model [48], [49], and the lack of encapsulated hypoxic lesions we observed in lung sections, we believe that the consequences of the phenotype reported here would be even more pronounced in models that more closely resemble human pathology, such as the rabbit or guinea pig.

The niche in which latent *M. tuberculosis* survives, avoiding immune surveillance and maintaining undetectable cell numbers, is presently unknown. Several hypotheses have been suggested including the necrotic centers of granulomas [50], [51], adipocytes [52], and recently mesenchymal stem cells [53]. This latter work is especially interesting in light of the conclusions presented here. It implies that the oxidative burst experienced when invading *M. tuberculosis* is engulfed by an alveolar macrophage would serve to inhibit respiration by shifting redox balance – toward an oxidized quinone pool in which quinol becomes limiting for oxygen reduction. Since cells in NRP-2 maintain an energized membrane, and are notably tolerant to single antibiotics but retain sensitivity to some combinations [54], we think it is plausible that persistence is a function of the oxidative state of the milieu, and is the result of reduced respiratory flux. The presence of adequate oxygen alone is not sufficient to stimulate respiration; quinone redox homeostasis must be restored before respiration can reach optimal levels and the cell can take advantage of the energetic benefit of oxygen as its terminal electron acceptor.

The necessity for members of the *M. tuberculosis* complex to maintain two possible *frd* enzymes (*sdhCDAB* & *frdABCD*) may be an indication of a metabolic plasticity which enables them to simultaneously utilize multiple carbon sources with different oxidation states and divert this carbon either into biomass production or storage molecules during growth, or into energy production for maintenance of PMF and repair during non-growth states [55], [56]. The previously observed rerouting of a portion of carbon flux into the reductive C4 arm of the TCA cycle [7] suggests the involvement of fumarate reductase activity in hypoxia, indicating that other pathways contribute to anaerobic survival to some extent. Redundancy in Frd catalysis remains a possible explanation, and further genetic analysis will need to be performed to establish this, but thus far we have been unable to delete both *sdh1* and *sdh2* (or *sdh2* & *frdABCD*) sequentially to address this hypothesis. Interestingly, Baek and Sassetti found that transposon mutagenesis of *sdh2* led to an inability to shut down growth in hypoxia, thus if the primary means of succinate oxidation is through Sdh1, oxygen (but not carbon) limitation does not result in cessation of growth, and succinate dehydrogenase activity continues to push carbon through the TCA cycle to continue biomass production [57].

There is now widespread acknowledgement of the fact that a reduction in the duration of TB chemotherapy could be achieved by finding ways to target non-replicating *M. tuberculosis*. The recent FDA approval of Bedaquiline lends credence to the idea that non-replicating cells still remain susceptible to inhibitors targeting maintenance bioenergetics, albeit at a reduced rate compared to current effective drugs [8], [58]–[60]. In this communication, we propose that the removal of a metabolic block on *M. tuberculosis* respiration imposed by the contending action of the aerobic succinate dehydrogenase and fumarate reductase activities would prevent the orderly metabolic shift to quiescence. Compounds that serve to reduce quinones in non-dividing organisms would exhibit the pleiotropic effects garnered by increasing respiration, including enhancing membrane potential-driven uptake and decreasing fitness. Thus, progress toward the goal of shortening chemotherapy might be better served by searching for *enhancers* of respiration, which may reduce the numbers of organisms which are shifted to a persistent state.

## Materials and Methods

### Mycobacterial strains and growth conditions

Attenuated strains *of M. tuberculosis* were constructed by allelic exchange via specialized transduction [15] from the parental strain H37Rv. Null mutants in *M. tuberculosis* strains H37Rv, mc^2^7000/mc^2^6230 (Δ*panCD*, ΔRD-1) [61], show identical growth characteristics in standard atmosphere as the parental strain (unpublished results). T-Coffee [62] was used to assess homology between enzyme subunits (Figure 1) and scores are presented as alignments of individual subunits corresponding to *sdh2*. For a full list of strains used in this work, see (Table S1). For CFU experiments, mycobacteria were grown to OD_600_ 0.5 and subcultured into media containing antibiotic and incubated at 37°C in a shaking incubator, or shifted to an anaerobic chamber (<1 ppm O_2_) in bottles with vented caps and incubated shaking at 37°C. For growth experiments using single carbon sources, 7H9 media was supplemented with NaCl and BSA and individual carbon sources (see Supplementary Methods for more detail).

### Metabolomics

Analysis was performed using an Acquity UPLC system (Waters, Manchester, UK) coupled with a Synapt G2 quadrupole–time of flight hybrid mass spectrometer. Column eluents were delivered via Electrospray Ionization. UPLC was performed in HILIC mode gradient elution using an Acquity amide column 1.7 µm (2.1×150 mm) using a method previously described [63]. The flow rate is 0.5 mL/min with mobile phase A (100% acetonitrile) and mobile phase B (100% water) both containing 0.1% formic acid. The gradient in both positive and negative mode is 0 min, 99% A; 1 min,99% A; 16 min, 30% A; 17 min, 30% A; 19 min 99% A; 20 min 99% A. The mass spectrometer was operated in V mode for high sensitivity using a capillary voltage of 2 kV and a cone voltage of 17 V. The desolvation gas flow rate is 500L/h, and the source and desolvation gas temperature are 120 and 325°C. MS spectra were acquired in centroid mode from m/z 50 to 1,000 using a scan time of 0.5 s. Leucine enkephalin (2 ng/µL) was used as lock mass (m/z 556.2771 and 554.2615 in positive and negative experiments, respectively). For further details, see Metabolomics in Text S1.

### Measurement of respiration

Measurement of oxygen consumption rate in *M. tuberculosis* was performed using a Clark-type oxygen electrode (Rank Brothers Cambridge, UK) with data collected using an ADC-24 data logger (Pico Technology, Cambridgeshire, UK). Cells were prepared in 490 cm^2^ roller bottles (HSR = 26), (Corning, NY). For culture densities below OD_600_ 4.0, cultures were centrifuged for 5 minutes at 4,000 rpm and resuspended in fresh 7H9 media from which catalase was omitted. To detect induction of oxygen consumption by reductants, 5 mL early stationary phase cells (OD_600_ 5.0) were added to the incubation chamber and basal oxygen consumption was monitored for 100–200 seconds, at which point compound was added. After 200 seconds, maximal uncoupled oxygen consumption rate was determined by the addition of 20 µM CCCP for 100 seconds.

### Mouse infections

We grew mycobacterial strains as described above in media containing OADC and the appropriate antibiotic for two passages before a single passage in media in which antibiotic was omitted immediately prior to animal infection. Female C57BL/6 mice, Rag-1^−/−^, and C3HeB/FeJ mice were obtained from Jackson Laboratory. Rag-1^−/−^ mice were infected with ∼1×10^6^ CFU of virulent mycobacteria via high volume tail vein injection. C57BL/6 mice and C3HeB/FeJ mice were infected via aerosol from a suspension of bacterial culture in PBS containing 0.05% Tween 80 and 0.004% antifoam, which yielded ≈100 or ≈50 cfu per lung. Four mice from each infection group were killed 24 h post-exposure, and lung homogenates were plated on 7H9-agar plates to determine the efficiency of aerosolization. We determined bacterial loads in lungs and spleen by plating for CFU at the indicated times from four mice per infection group. Five mice from each group were also used to determine survival times of infected mice. Pathological analysis and histological staining of organ sections were done on tissues fixed in buffered 10% formalin. Mouse protocols used in this work were approved by the Institutional Animal Care and Use Committee of Albert Einstein College of Medicine.

### Ethics statement

Mouse studies were performed in accordance to National Institutes of Health guidelines using recommendations in the Guide for the Care and Use of Laboratory Animals. The protocols used in this study were approved by the Institutional Animal Care and Use Committee of Albert Einstein College of Medicine (Protocol #20120114)

### Accession numbers

NP_214761 (Rv0247c), AFN48101 (Rv0248c), CCP42978 (Rv0249c), CCP46136 (SdhC), CCP46137 (SdhD), CCP46138 (SdhA), CCP46139 (SdhB), NP_216370 (Ndh), CCP43122 (NdhA)

## Supporting Information

Figure S1 **Deletion of** ***sdh1*** **results in succinate accumulation in both virulent and attenuated** ***M. tuberculosis*** **strains** *M. tuberculosis* intracellular metabolite concentrations were performed as previously described. Parental or mutant cultures were grown to OD_600_ 0.5 (aerobic) and metabolites were extracted (see supplemental methods), Data is reported as mean peak intensity ±SEM adjusted to OD_600_ for three replicate experiments from parental strain (H37Rv), Δsdh1 (RvΔsdh1), Δsdh2 (RvΔsdh2), Δsdh1::pYUB1738 (P_hsp60_:sdh1), or Δsdh2::pYUB1737 (P_hsp60_:sdh2), strains. Statistical significance determined by ANOVA, *, p<0.05; ****, p<0.0001.
(TIF)

Figure S2 **Stable isotope labeling confirms Sdh1 to be an aerobic succinate dehydrogenase.** Direction of carbon flux was determined by addition of 1,4 ^13^C_2_-aspartate to cells in mid logarithmic growth phase (A) or after 10 days of hypoxic adaptation (B) and extraction following a 24 hour labeling period (see Metabolomics in Text S1). The diagram depicts the proportion of relevant isotopologues for each intermediate on a simplified TCA schematic. Fold-change was calculated by determination of the labeled proportion of each isotopomer consisting of labeled intensities minus weighted average intensities to normalize for naturally occurring isotopes, then divided by the sum of labeled intensities. This value represents the proportion (P_lab__mutant) of each isotopomer in comparison with the parental strain (mc^2^6230) (P_lab__ *wt*). Rows show fold-differences of labeled metabolites, adjusted for cell density (OD_600_) and corrected for natural isotope abundance, for Δ*sdh1* mutant (top row) or Δ*sdh2* mutant (bottom row) with respect to the parental strain. Arrows within cells indicate increase, decrease, or no change in abundance for respective isotopologues of mean intensity from three biological replicates and are meant to be illustrative.
(TIF)

Figure S3 **Sdh mutants display decreased survival in hypoxia upon disruption of the proton gradient.** Viability in aerobic (A) and anaerobic (B) conditions were examined by disruption of the proton gradient (CCCP –10 µM) (see Methods) and plating CFUs at timepoints indicated. The results from a single representative experiment are shown here.
(TIF)

Figure S4 **Regulated expression is desirable for complementation of ETC gene deletions.** Complementation of the respiratory phenotype of *M. tuberculosis* Δ*sdh1* requires low levels of induction for minimal expression. (A) To remedy the overcomplementation of Sdh1 observed when *Rv0249c-Rv0247c* are expressed using an integrative vector (pMV361 - containing P_hsp60_), an inducible plasmid (pYUB1734) was constructed and was designated pYUB1753 (P_tet2_:*sdh1*) or pYUB1754 (P_tet2_:*sdh2*). Parent and mutant strains were inoculated into a bioreactor at OD_600_ 0.05 in 7H9 complete media containing 25 ng/ml anhydrotetracycline and allowed to respire available oxygen (see Complementation in Text S1 for details). Over-complementation of mc^2^6230 Δ*ndhA* (B) by an integrated constitutive promoter (P_hsp60_) illustrates the tuning of quinone redox balance to maintenance of respiratory rate. Strains mc^2^6230, Δ*ndhA* (mc^2^5872), and Δ*ndhA* complemented (P_hsp60_:*ndhA* - mc^2^5874) were grown in batch culture bioreactors and allowed to consume available oxygen. DO was recorded as described in Methods.
(TIF)

Figure S5 **Increased respiration of Δ** ***sdh1*** **does not result in increased growth rate in batch culture experiments.** Growth of *M. tuberculosis* strains was monitored during batch culture using an absorbance probe at OD_600_. X-axis (Inoculation time) includes data only from the time during which oxygen is available (first 8 days), absorbance is measured at intervals and values are interpolated for visualization.
(TIF)

Figure S6 **Changes in midpoint redox potential of cultures operating in batch mode** ***precede*** **resumption of respiration.** The parental strain of mc^2^6230 was inoculated into a bioreactor configured to sparge 4-6 L/hr. air once DO tension dropped below 1%. Midpoint redox potential (red line) was measured concurrently with DO (blue line) using a separate redox probe (DasGip, Jülich, Germany) with readings every 30 s. As oxygen is depleted, cells are seen to switch off respiration and DO builds up in the vessel. A change in redox midpoint potential (red shaded) can be seen before oxygen consumption resumes (blue shaded) and the process repeats twice more. Cell density was OD_600_>1.0 over the days depicted here. Data is representative of two separate experiments.
(TIF)

Figure S7 **ΔpH during aerobic growth.** Change in pH was observed in batch culture media over the course of aerobic growth for *M. tuberculosis* mutant strains in bioreactors operated in batch mode (see Methods). pH meters are calibrated to standards prior to each experiment.
(TIF)

Figure S8 **Δsdh1 maintains a negative redox potential in depleted oxygen conditions.** DO consumption (top panel) and redox potential (bottom panel) of mc^2^6230, Δ*sdh1*, and complemented strain were monitored in batch culture in a controlled bioreactor set to maintain 1%DO by sparging air (see Figure S7 and Methods in Text S1).
(TIF)

Figure S9 **Sdh mutants are not attenuated in C57Bl/6 mice.** Mice were infected via low dose aerosol (see Methods in Text S1) and burden was assessed over time by plating whole organ homogenates on 7H10 plates. Colonies were counted after incubation for 3–4 weeks at 37°C. Four mice per group were sacrificed at each timepoint.
(TIF)

Figure S10 **Lung pathology from C3HeB/FeJ mice does not indicate encased lung lesions.** Mice were infected as described in Methods. At twenty weeks post-infection, the left lung was harvested from 4 mice per group, paraffin embedded, and fixed in 2% paraformaldehyde prior to sectioning. Whole lungs were sectioned and alternate sections were acid fast and hematoxylin and eosin stained. The images above were chosen to be representative of each group.
(TIF)

Text S1 **Supporting information and tables.**
(DOCX)
