# Supplementary figures and images for: Succinate Dehydrogenase is the Regulator of Respiration in Mycobacterium tuberculosis
Source: PLoS Pathog. 2014 Nov 20;10(11):e1004510. doi: 10.1371/journal.ppat.1004510 (PMC4239112; doi:10.1371/journal.ppat.1004510)

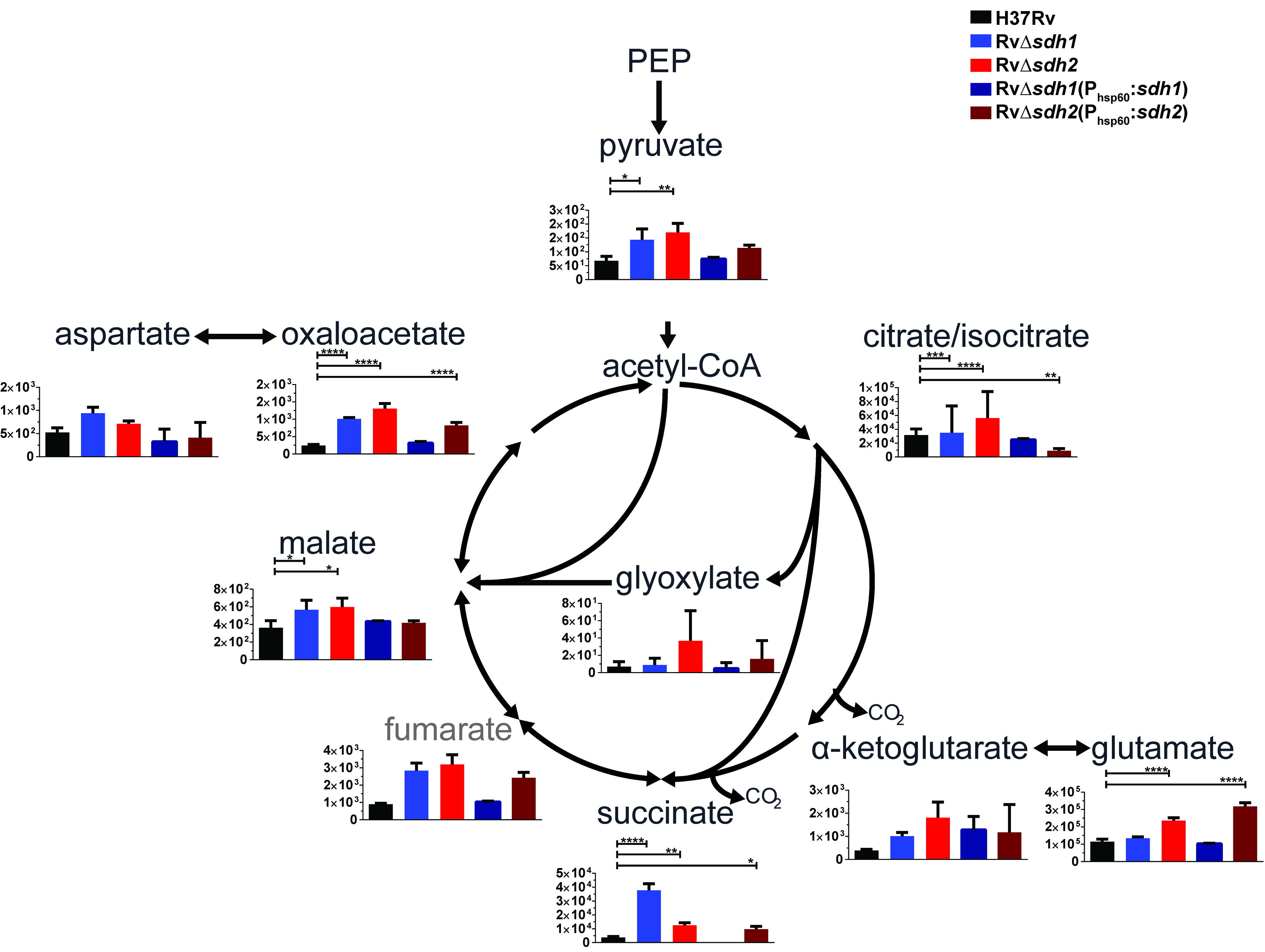

Supplement: Figure S1 — Deletion of sdh1 results in succinate accumulation in both virulent and attenuated M. tuberculosis strains M. tuberculosis intracellular metabolite concentrations were performed as previously described. Parental or mutant cultures were grown to OD600 0.5 (aerobic) and metabolites were extracted (see supplemental methods), Data is reported as mean peak intensity ±SEM adjusted to OD600 for three replicate experiments from parental strain (H37Rv), Δsdh1 (RvΔsdh1), Δsdh2 (RvΔsdh2), Δsdh1::pYUB1738 (Phsp60:sdh1), or Δsdh2::pYUB1737 (Phsp60:sdh2), strains. Statistical significance determined by ANOVA, *, p<0.05; ****, p<0.0001. (TIF) [file ppat.1004510.s001.tif]

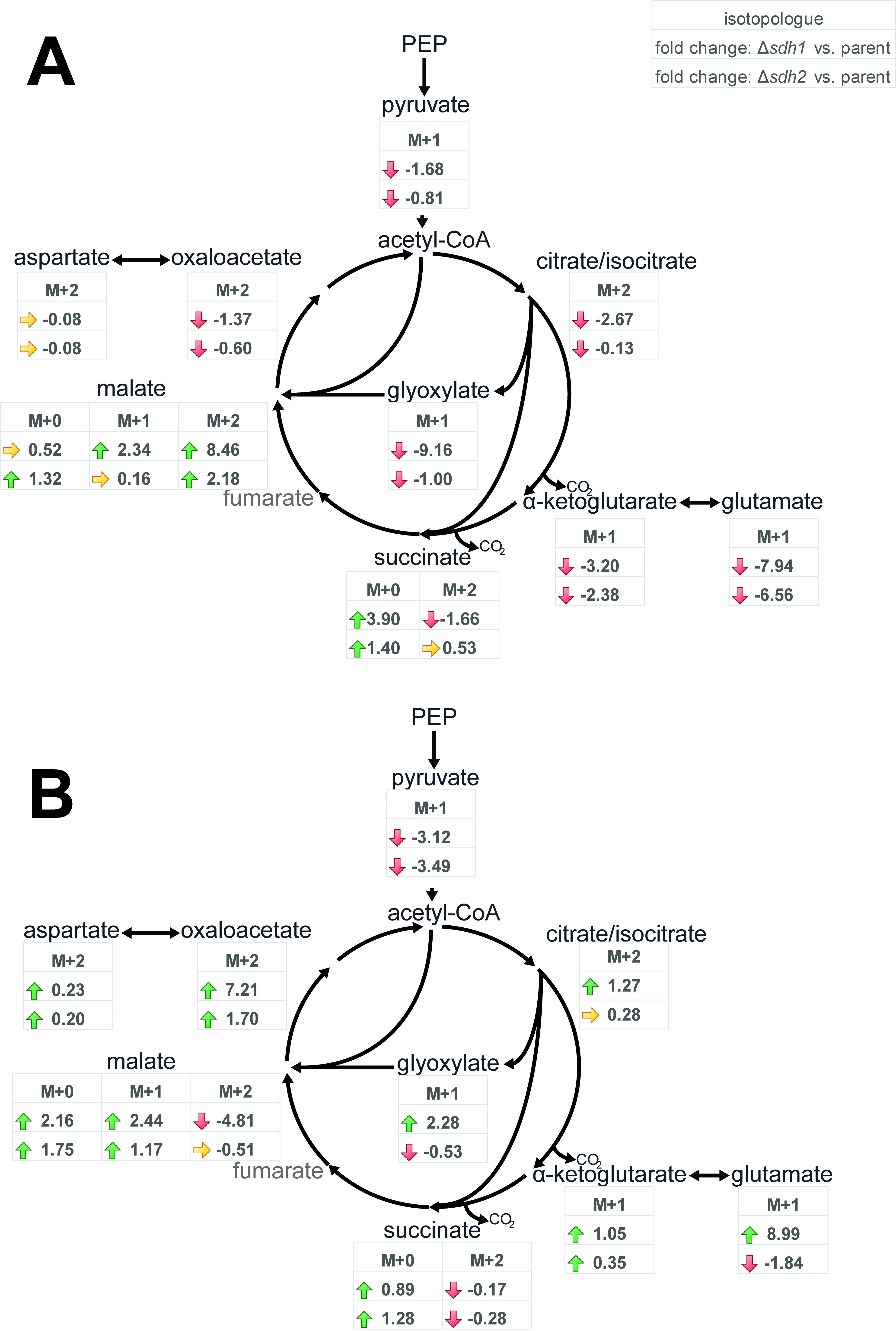

Supplement: Figure S2 — Stable isotope labeling confirms Sdh1 to be an aerobic succinate dehydrogenase. Direction of carbon flux was determined by addition of 1,4 13C2-aspartate to cells in mid logarithmic growth phase (A) or after 10 days of hypoxic adaptation (B) and extraction following a 24 hour labeling period (see Metabolomics in Text S1). The diagram depicts the proportion of relevant isotopologues for each intermediate on a simplified TCA schematic. Fold-change was calculated by determination of the labeled proportion of each isotopomer consisting of labeled intensities minus weighted average intensities to normalize for naturally occurring isotopes, then divided by the sum of labeled intensities. This value represents the proportion (Plab_mutant) of each isotopomer in comparison with the parental strain (mc26230) (Plab_ wt). Rows show fold-differences of labeled metabolites, adjusted for cell density (OD600) and corrected for natural isotope abundance, for Δsdh1 mutant (top row) or Δsdh2 mutant (bottom row) with respect to the parental strain. Arrows within cells indicate increase, decrease, or no change in abundance for respective isotopologues of mean intensity from three biological replicates and are meant to be illustrative. (TIF) [file ppat.1004510.s002.tif]

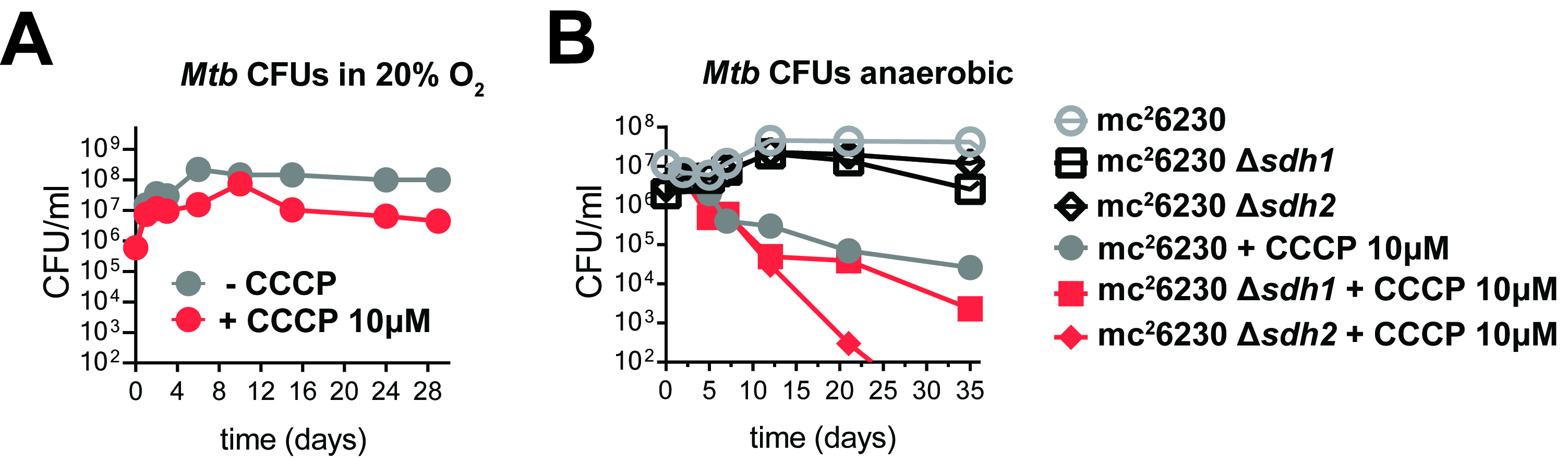

Supplement: Figure S3 — Sdh mutants display decreased survival in hypoxia upon disruption of the proton gradient. Viability in aerobic (A) and anaerobic (B) conditions were examined by disruption of the proton gradient (CCCP –10 µM) (see Methods) and plating CFUs at timepoints indicated. The results from a single representative experiment are shown here. (TIF) [file ppat.1004510.s003.tif]

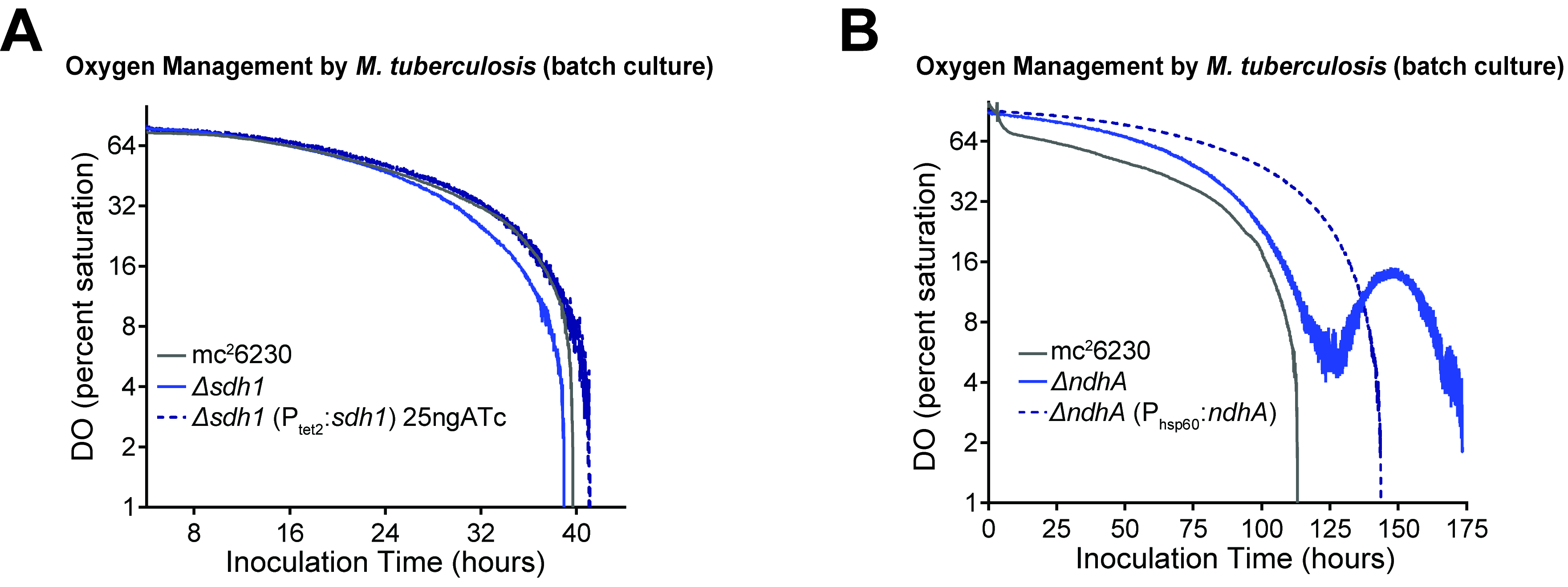

Supplement: Figure S4 — Regulated expression is desirable for complementation of ETC gene deletions. Complementation of the respiratory phenotype of M. tuberculosis Δsdh1 requires low levels of induction for minimal expression. (A) To remedy the overcomplementation of Sdh1 observed when Rv0249c-Rv0247c are expressed using an integrative vector (pMV361 - containing Phsp60), an inducible plasmid (pYUB1734) was constructed and was designated pYUB1753 (Ptet2:sdh1) or pYUB1754 (Ptet2:sdh2). Parent and mutant strains were inoculated into a bioreactor at OD600 0.05 in 7H9 complete media containing 25 ng/ml anhydrotetracycline and allowed to respire available oxygen (see Complementation in Text S1 for details). Over-complementation of mc26230 ΔndhA (B) by an integrated constitutive promoter (Phsp60) illustrates the tuning of quinone redox balance to maintenance of respiratory rate. Strains mc26230, ΔndhA (mc25872), and ΔndhA complemented (Phsp60:ndhA - mc25874) were grown in batch culture bioreactors and allowed to consume available oxygen. DO was recorded as described in Methods. (TIF) [file ppat.1004510.s004.tif]

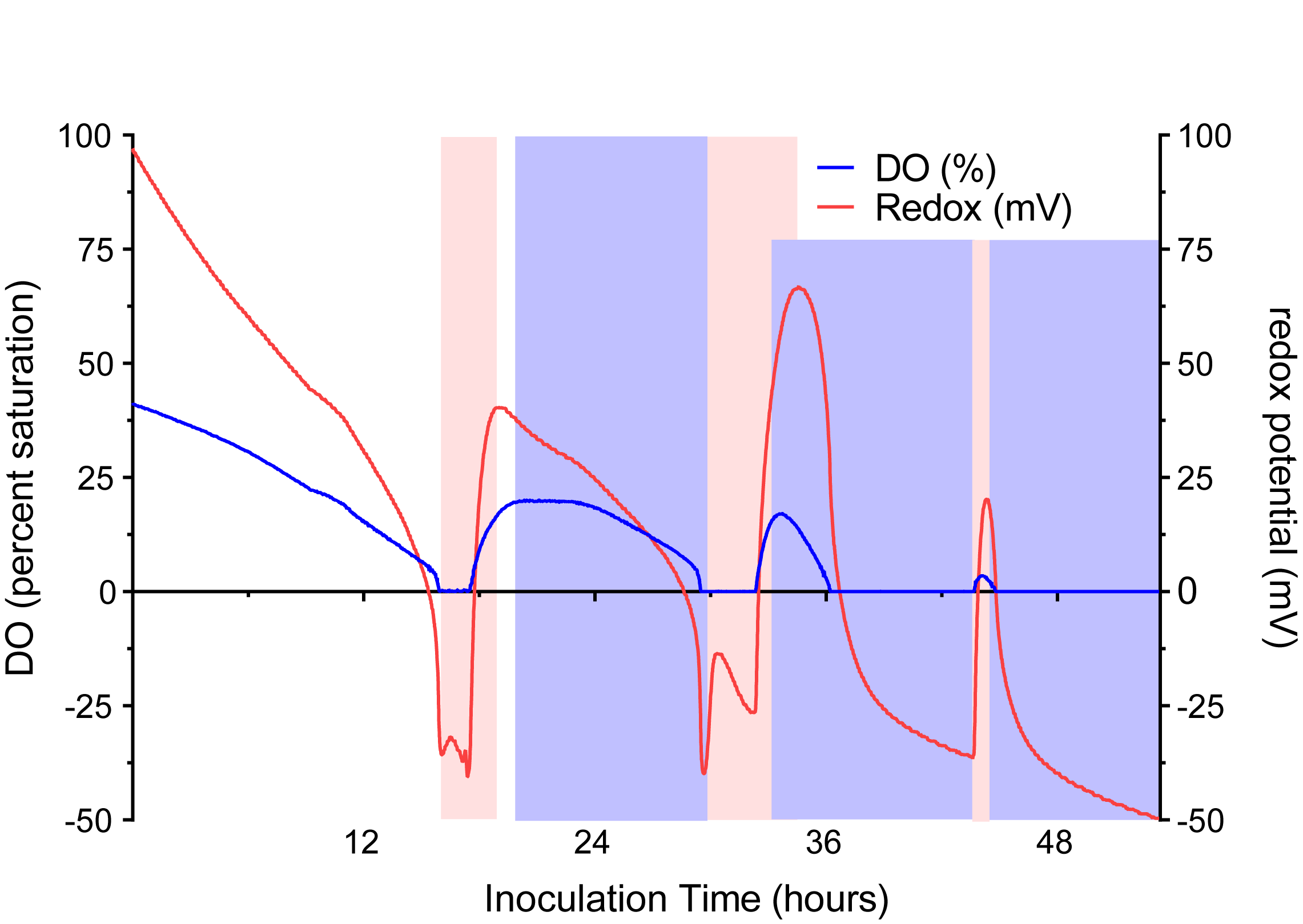

Supplement: Figure S6 — Changes in midpoint redox potential of cultures operating in batch mode precede resumption of respiration. The parental strain of mc26230 was inoculated into a bioreactor configured to sparge 4-6 L/hr. air once DO tension dropped below 1%. Midpoint redox potential (red line) was measured concurrently with DO (blue line) using a separate redox probe (DasGip, Jülich, Germany) with readings every 30 s. As oxygen is depleted, cells are seen to switch off respiration and DO builds up in the vessel. A change in redox midpoint potential (red shaded) can be seen before oxygen consumption resumes (blue shaded) and the process repeats twice more. Cell density was OD600>1.0 over the days depicted here. Data is representative of two separate experiments. (TIF) [file ppat.1004510.s006.tif]

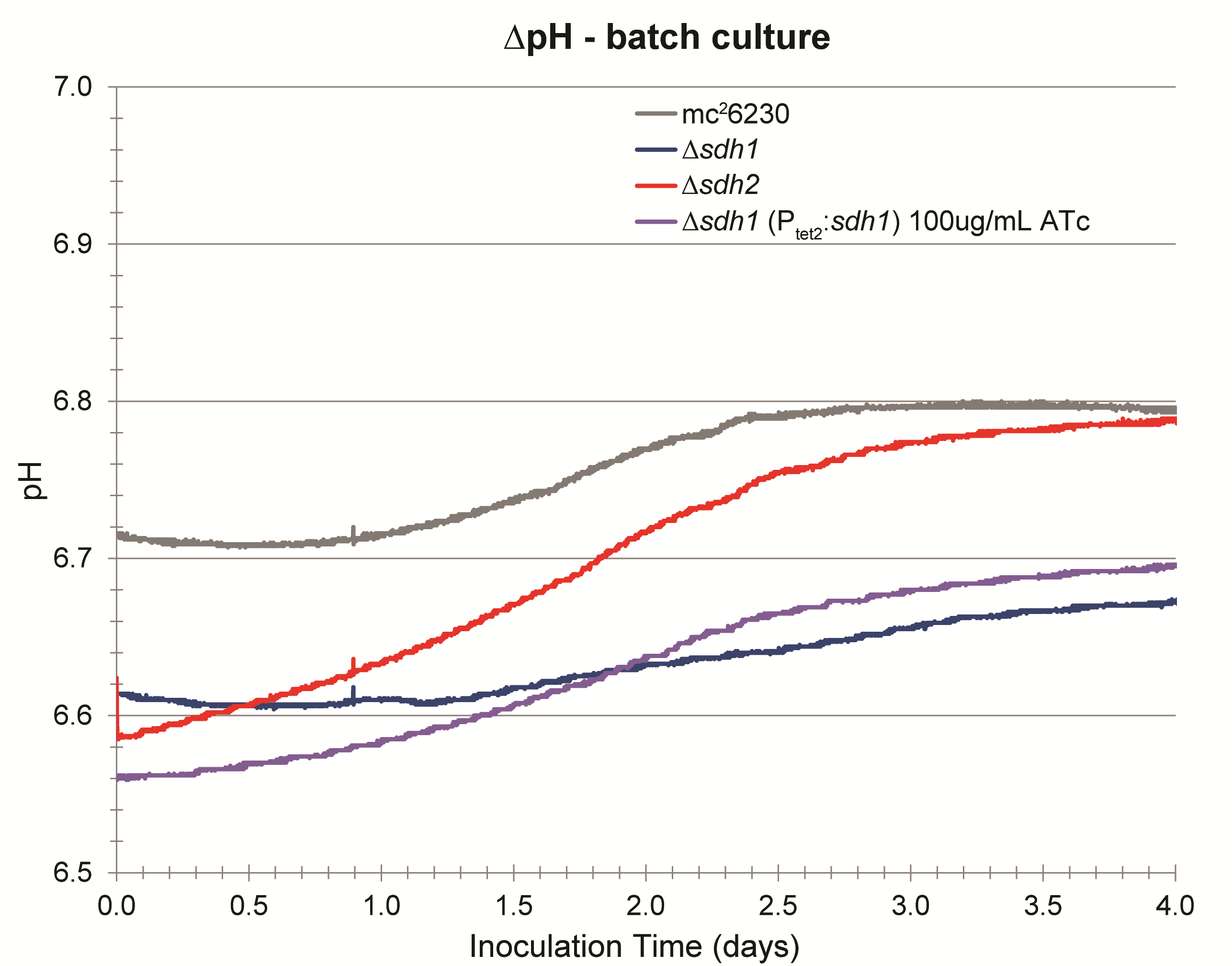

Supplement: Figure S7 — ΔpH during aerobic growth. Change in pH was observed in batch culture media over the course of aerobic growth for M. tuberculosis mutant strains in bioreactors operated in batch mode (see Methods). pH meters are calibrated to standards prior to each experiment. (TIF) [file ppat.1004510.s007.tif]

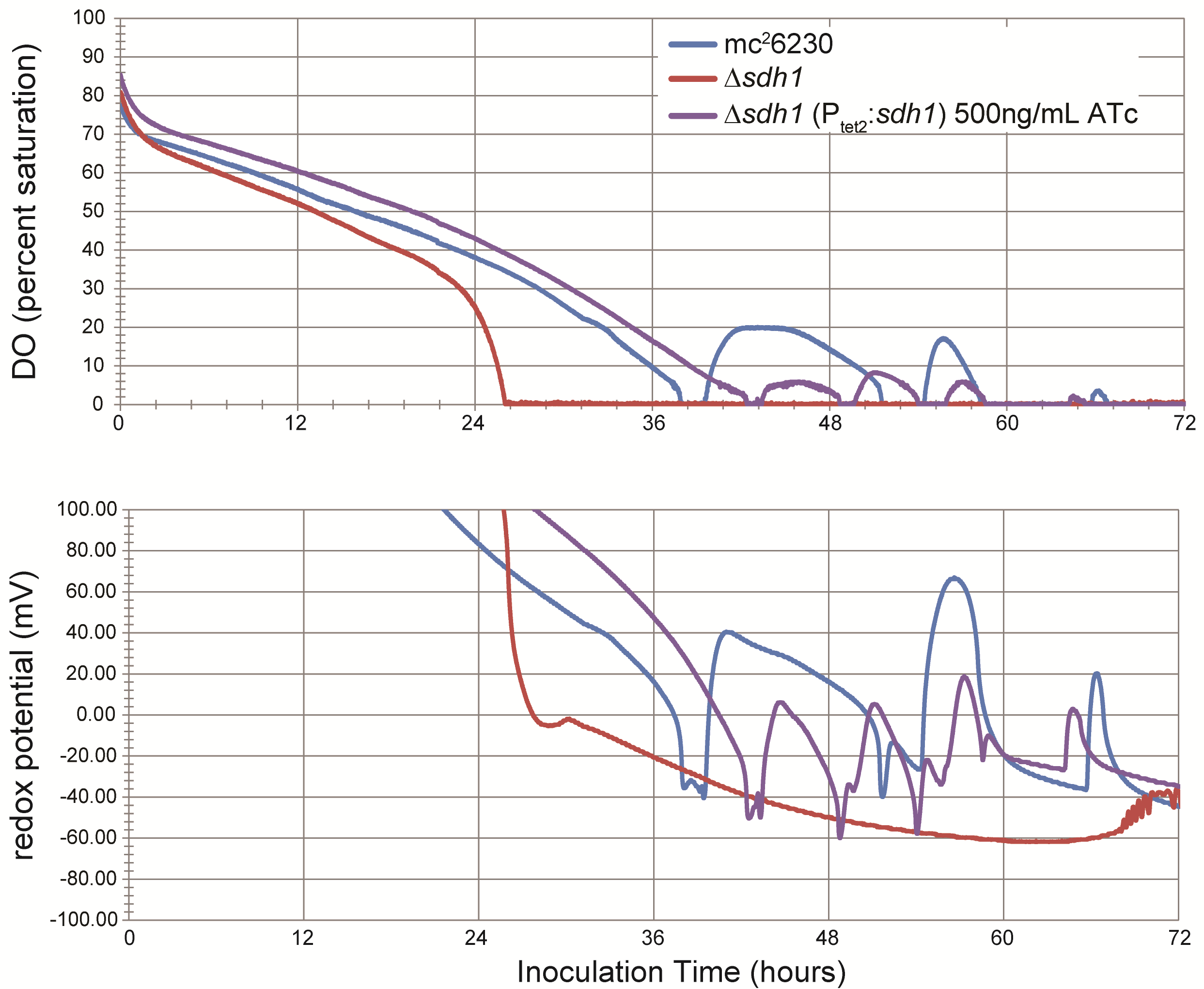

Supplement: Figure S8 — Δsdh1 maintains a negative redox potential in depleted oxygen conditions. DO consumption (top panel) and redox potential (bottom panel) of mc26230, Δsdh1, and complemented strain were monitored in batch culture in a controlled bioreactor set to maintain 1%DO by sparging air (see Figure S7 and Methods in Text S1). (TIF) [file ppat.1004510.s008.tif]

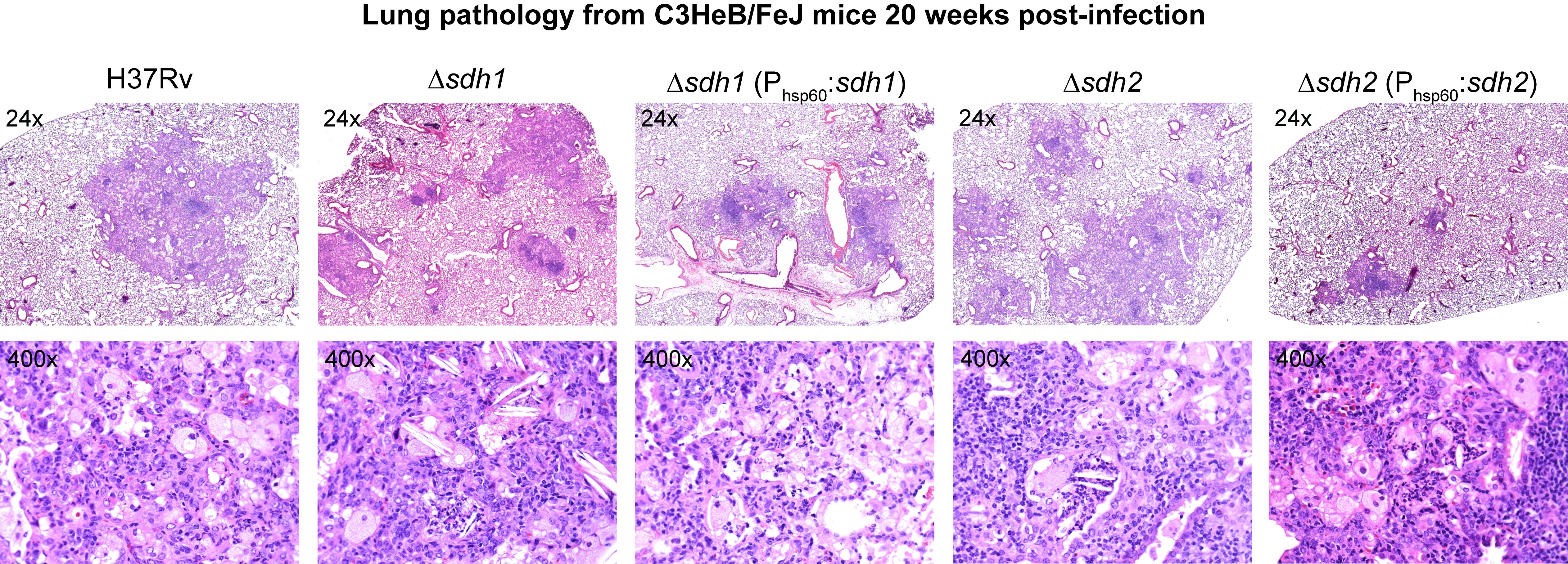

Supplement: Figure S10 — Lung pathology from C3HeB/FeJ mice does not indicate encased lung lesions. Mice were infected as described in Methods. At twenty weeks post-infection, the left lung was harvested from 4 mice per group, paraffin embedded, and fixed in 2% paraformaldehyde prior to sectioning. Whole lungs were sectioned and alternate sections were acid fast and hematoxylin and eosin stained. The images above were chosen to be representative of each group. (TIF) [file ppat.1004510.s010.tif]
