# Supplementary material for: Succinate Dehydrogenase is the Regulator of Respiration in Mycobacterium tuberculosis
Source: PLoS Pathog. 2014 Nov 20;10(11):e1004510. doi: 10.1371/journal.ppat.1004510 (PMC4239112; doi:10.1371/journal.ppat.1004510)
Supplement: Text S1 — Supporting information and tables. (DOCX) [file ppat.1004510.s011.docx]

# Supporting Text:

### Materials and Methods

#### Mycobacterial strains and growth conditions

Attenuated strains *of M. tuberculosis* were constructed by allelic exchange via specialized transduction [1] from the parental strain H37Rv. Null mutants in *M. tuberculosis* strains H37Rv, mc^2^7000 / mc^2^6230 (Δ*panCD*, ΔRD-1)[2], show identical growth characteristics in standard atmosphere as the parental strain (unpublished results). T-Coffee [3] was used to assess homology between enzyme subunits (Figure 1) and scores are presented as alignments of individual subunits corresponding to *sdh2*. For a full list of strains used in this work, see (Table S1). For CFU experiments, mycobacteria were grown to OD_600_ 0.5 and subcultured into media containing antibiotic and incubated at 37ºC in a shaking incubator, or shifted to an anaerobic chamber (<1 ppm O_2_) in bottles with vented caps and incubated shaking at 37ºC (see Supplementary Methods for more detail).

For growth experiments, cells were grown in Middlebrook 7H9 (Difco, Sparks, MD) supplemented with Middlebrook OADC Growth Supplement (Difco, Sparks, MD), glycerol (5 mg/mL) and tyloxapol (0.5 mg/ml). For single carbon source growth curves, OADC was omitted and replaced with NaCl (0.85 mg/ml) and bovine albumin-fraction V (5 mg/mL) plus the carbon source of interest. Media was carbon balanced for single carbon source experiments to ensure total carbon availability was constant across conditions. For auxotrophic strains, pantothenic acid (50 µg/ml) was added.

For experiments requiring growth conditions involving low oxygen, cells were incubated in a controlled atmosphere chamber (Coy Laboratory Products, Grass Lake, MI), fitted with an AC100 CO_2_ controller (Coy Laboratory Products). The internal atmosphere was composed of 4.96% CO_2_, 5.99% H_2_, and the balance Nitrogen as reported previously (Baughn et al., 2009).

For controlled batch culture experiments, DasGip mini bioreactors were employed (DasGip, Jülich, Germany). A 24hr calibration of oxygen probes and pH probes was performed in vessels containing 200ml of sterile media prior to inoculation for each experiment. Except where otherwise noted (Figure S4A), inoculation was performed at OD_600_ 0.005. Culture volumes were 200ml stirring at 100rpm for batch culture or cascaded to DO level for experiments with fixed O_2_ concentration.

For chemostat experiments, the same bioreactors were used. *M. tuberculosis* strains were inoculated into bioreactors containing 200 mL media and continuous culture conditions were initiated by feeding 2 mL/hr fresh media to maintain a specific growth rate (μ) of 0.01. Gassing and stir rate were cascaded to the DO process value (50% DO_2_ initial), and once steady state conditions were reached, cells were maintained at that DO_2_ value for 24 hours before sampling (note dips in absorbance (AU) at sampling times). After sampling, DO_2_ process values were shifted downward (50% to 25% to 5% to 1%) in steps.

For TraSH, a library of ~10^6^ independent mutants were generated using phAE180 (a mariner-based transposon delivery phage[4]) Mutant strains were pooled in 7H9 medium and split to OD_600_ 0.1 into roller bottles containing 100 mL 7H9 medium supplemented with ADS, glycerol, and Tween80 (see Methods) ± INH 1 µg/ml RIF 5 µg/ml and incubated four days. Cells were then pelleted and genomic DNA was extracted. This experiment was repeated from two independent transposon pools with at least three independent biological replicates each. TraSH was performed according to the published protocol [5], with the exception that cells were not plated and re-grown post-treatment. Probes were made and hybridized to DNA microarrays obtained from the US National Institute of Allergy and Infectious Diseases’ Pathogen Functional Genomics Resource Center (PFGRC). Hits were deemed significant by t-test at Alpha (overall threshold p-value) of 0.01.

Antibiotics, authentic standards for mass spectroscopy, and uncoupling agents were purchased from Sigma Aldrich, St. Louis, MO. Fluorescent ROS dyes were purchased from Molecular Probes, Grand Island, NY.

#### Metabolomics

For metabolomic experiments, cells were grown in Middlebrook 7H9 and plated on 7H10 media (Difco, Sparks, MD) supplemented with Middlebrook OADC Growth Supplement (Difco, Sparks, MD), glycerol (5 mg/mL) and tyloxapol (0.5 mg/ml). For antibiotic treatment, OADC was omitted and replaced with NaCl (0.85 mg/ml) dextrose (2 mg/ml), and bovine albumin-fraction V (5 mg/mL). For auxotrophic strains, pantothenic acid (50 µg/ml) was added. Cultures were grown in 490cm^2^ roller bottles (HSR =26), (Corning, NY) with agitation at 100rpm and 37°C or in 30 ml inkwell bottles (Nalgene Rochester, NY) for routine culture. Bioreactor experiments were performed in a DASBOX parallel bioreactor system from DASGIP (Eppendorf, USA) with 200 ml working volume. Cell density, CFU/mL, and protein mg/mL were within a 2-fold range for samples at time of extraction.

Analysis was performed using an Acquity UPLC system (Waters, Manchester, UK) coupled with a Synapt G2 quadrupole–time of flight hybrid mass spectrometer. Column eluents were delivered via Electrospray Ionization. UPLC was performed in HILIC mode gradient elution using an Acquity amide column 1.7 μm (2.1 × 150 mm) using a method previously described [6]. The flow rate is 0.5 mL/min with mobile phase A (100% acetonitrile) and mobile phase B (100% water) both containing 0.1% formic acid. The gradient in both positive and negative mode is 0 min, 99% A; 1 min,99% A; 16 min, 30% A; 17 min, 30% A; 19 min 99% A; 20 min 99% A. The mass spectrometer was operated in V mode for high sensitivity using a capillary voltage of 2 kV and a cone voltage of 17 V. The desolvation gas flow rate is 500L/h, and the source and desolvation gas temperature are 120 and 325°C. MS spectra were acquired in centroid mode from m/z 50 to 1,000 using a scan time of 0.5 s. Leucine enkephalin (2 ng/μL) was used as lock mass (m/z 556.2771 and 554.2615 in positive and negative experiments, respectively).

Data was analyzed using MassLynx and TargetLynx software (Waters, Manchester, UK). All reported metabolites were verified using authentic standards and the UPLC gradient described above. Data is reported as total peak intensity normalized to optical density of the sampled culture at 600 nM, and minus background subtraction or as proportion of labeled metabolite corrected for natural isotope abundance and with respect to parental strain under the same condition.

For aqueous metabolite pools, hypoxic and aerobically growing cultures were harvested in log phase (OD_600_ 0.6-0.7) or after 12 days of incubation in a controlled atmosphere chamber (Coy Laboratory Products, Grass Lake, MI) with 0% O_2_, 5% CO_2_, 10% H_2_, balance nitrogen. 100 mL cells were grown in 490 cm^2^ roller bottles (head space ratio – HSR 13.5:1) (Corning Inc. Corning, NY) at 37ºC to OD_600_ 0.5 and shifted to controlled atmosphere, 5 mL cell cultures (per timepoint) were rapidly quenched in 10 mL 100% MeOH at -20°C and centrifuged at 4000 rpm for 10 min at -9°C. Cell pellets were resuspended in Extraction solvent containing 40% ACN, 40% MeOH, 20% H_2_O and transferred to tubes containing silica beads then agitated 2x in a FastPrep-24 (MP Bio, Solon, OH) with 5min on ice between beatings. Samples were briefly spun and 750 µL of extract was filtered through a sterile 0.22 µm filter, and then frozen at -80°C until time of analysis. Extracts were made in triplicate Data is reported as total peak intensity for each exact mass (± 0.05 Da) adjusted to OD_600_ of individual samples and integrated over a retention time window based on UPLC elution in an identical gradient from an authentic standard (Sigma Aldrich, St. Louis, MO). Where applicable, standards were spiked into TB lysates to test for suppression effects. Statistics were performed with Prism (GraphPad Software, Inc., La Jolla, CA) for ANOVA on mean intensity differences, or MarkerLynx (Waters, Manchester, UK), for principal component analysis (PCA).

For isotope labeling experiments the method of Watanabe et. al. was followed. Hypoxic and aerobically growing cultures were labeled with [1, 4-^13^C2] L-aspartic acid or [U-13C] L-aspartic acid. 187.5 µL of 20 mg/mL ^13^C-sources were added into 1.5 mL of culture in triplicate and incubated for 20 or 24 h at 37°C. Post-incubation, cultures were rapidly quenched in methanol and aqueous metabolites were extracted using the method detailed above. Extracts were frozen at −80°C until time of analysis

For membrane potential measurements we followed the method of Zilberstein [7]. Briefly, hypoxic and aerobically growing cultures were harvested in log phase (OD=0.6-0.7) or after 10 days of incubation in a controlled atmosphere chamber. For the determination of ΔΨ, In these experiments, cells (1 ml) are taken directly from the fermenter/chemostat and added (quickly) to 5 ml polystyrene (or glass) test-tubes containing [^3^H]TPP+ (2.4 nM final concentration). After incubation for 5-10 min at 37°C, the reactions were stopped by the adding 2 ml cold 0.1 M LiCI and rapid filtration through 0.45-µM cellulose-acetate filter (Sartorius). Filters were dried and resuspended in scintillation fluid and counts were recorded on a MicroBeta Liquid Scintillation Counter (Perkin Elmer, Waltham, Massachusetts).

For MKH_2_/MK HPLC analysis, samples from batch cultures were taken by pipetting within 2 s. Samples (2 ml) were quenched with 6 ml ice-cold 0.2 M HClO_4_ in methanol. Petroleum ether (6 ml; 40–60 °C) was then added to the mixture, and vortexed for 1 min. After the mixture was centrifuged (900 g, 5 min), the upper petroleum ether phase was removed, transferred to a test tube, and dried under a flow of nitrogen. 6 ml petroleum ether was added to the lower phase, and the vortexing and centrifugation steps were repeated. The upper phases were combined. After second evaporation, extracts were stored at −80 °C. Immediately before use, the extracted MK was resuspended in 100 μl ethanol and analyzed in a HPLC system (Hewlett-Packard model HP1100 gradient chromatograph) coupled to a reverse-phase C18 column; Alltima C18 (4.6 mm i.d., 150 mm length, 3-μm column diameter) (Grace Discovery Sciences, Albany, OR ). The column was equilibrated with ethanol: methanol (1: 1, v/v), and this mixture was used as the mobile phase at a flow rate of 1 ml min^−1^. Detection was performed at 248 nm for MKs. The amounts of all quinones were calculated from the peak areas using a standard curve of MK-9 (Santa Cruz Biotechnology, Inc., Santa Cruz, CA), according to the method applied by Bekker [8]. Methanol, ethanol and petroleum ether were MS or analytical grade.

#### Measurement of respiration

Measurement of oxygen consumption rate in *M. tuberculosis* was performed using a Clark-type oxygen electrode (Rank Brothers Cambridge, UK) with data collected using an ADC-24 data logger (Pico Technology, Cambridgeshire, UK). Cells were prepared in 490 cm^2^ roller bottles (HSR =26), (Corning, NY). For culture densities below OD_600_ 4.0, cultures were centrifuged for 5 minutes at 4,000 rpm and resuspended in fresh 7H9 media from which catalase was omitted. To detect induction of oxygen consumption by reductants, 5 mL early stationary phase cells (OD_600_ 5.0) were added to the incubation chamber and basal O_2_ consumption was monitored for 100-200 seconds, at which point compound was added. After 200 seconds, maximal uncoupled oxygen consumption rate was determined by the addition of 20 µM CCCP for 100 seconds

#### Microarray Analysis

For transcriptional analysis, 10 mL of *M. tuberculosis* mc^2^6230, mc^2^7296, and mc^2^7297 cells were collected from either exponentially growing cultures (~OD_600_ 0.4 nm), or vented cultures incubated in an anaerobic atmosphere for 12 days (see Growth Conditions). Cell lysis and RNA preparation was performed according to protocols previously described [9]. RNA was fixed with Qiagen RNA Protect (Qiagen, Germantown, MD). Cells were disrupted by mechanical lysis and total RNA was purified using the Qiagen RNeasy kit. DNA hybridization was performed using arrays provided by the Pathogen Functional Genomics Research Center (PFGRC) from The Institute of Genomic Research (TIGR). TIGR Spotfinder was used to grid and quantitate hybridized cDNA and TIGR MIDAS was used for Lowess normalization, s.d. regularization and replicate analysis. Normalized results were analysed in TIGR MeV with Significance Analysis of Microarrays considered significant at q<0.05 Slides that had abnormal intensity profiles or that were below intensity threshold were rejected.

### Table S1. Strains and plasmids used in this work.

| **Strain number** | **parent** | **genotype/modification=(plasmid name)** |
| --- | --- | --- |
| **mc^2^2 (H37Rv)** | *-* | *RD1^+^ panCD^+^* |
| **mc^2^7000** | mc^2^2 | Δ*RD1* Δ*panCD* |
| **mc^2^6230** | mc^2^2 | Δ*RD1* Δ*panCD* |
| **mc^2^7292** | mc^2^2 | *RD1^+^ panCD^+^* Δ(*Rv0247c, Rv0248c, Rv0249c*) |
| **mc^2^7293** | mc^2^2 | *RD1^+^ panCD^+^* Δ*(sdhC, sdhD, sdhA, sdhB*) |
| **mc^2^7294** | mc^2^7292 | *RD1^+^ panCD^+^* Δ*(Rv0247c, Rv0248c, Rv0249c*) :: P_hsp60_*(Rv0247c, Rv0248c, Rv0249c*) =(pYUB1738) |
| **mc^2^7295** | mc^2^7293 | *RD1^+^ panCD^+^* Δ*(sdhC, sdhD, sdhA, sdhB) ::* P_hsp60_(*sdhC, sdhD, sdhA, sdhB*) =(pYUB1737) |
| **mc^2^7296** | mc^2^6230 | Δ*RD1* Δ*panCD* Δ*(Rv0247c, Rv0248c, Rv0249c)* |
| **mc^2^7297** | mc^2^6230 | *ΔRD1* Δ*panCD* Δ*(sdhC, sdhD, sdhA, sdhB)* |
| **mc^2^7298** | mc^2^7296 | Δ*RD1* Δ*panCD* Δ*(Rv0247c, Rv0248c, Rv0249c*) :: P_hsp60_(*Rv0247c, Rv0248c, Rv0249c*) =(pYUB1738) |
| **mc^2^7299** | mc^2^7297 | Δ*RD1* Δ*panCD* Δ*(sdhC, sdhD, sdhA, sdhB*) :: P_hsp60_(*sdhC, sdhD, sdhA, sdhB*) =(pYUB1737) |
| **mc^2^7300** | mc^2^7296 | Δ*RD1* Δ*panCD* Δ*(Rv0247c, Rv0248c, Rv0249c*) :: P_tet2_(*Rv0247c, Rv0248c, Rv0249c*) =(pYUB1757) |
| **mc^2^7301** | mc^2^7297 | Δ*RD1* Δ*panCD* Δ(*sdhC, sdhD, sdhA, sdhB*) :: P_tet2_(*sdhC, sdhD, sdhA, sdhB*) =(pYUB1756) |
| **mc^2^5871** | mc^2^6230 | Δ*RD1* Δ*panCD* Δ*ndh* |
| **mc^2^5873** | mc^2^6230 | Δ*RD1* Δ*panCD* Δ*ndh::*P_hsp60_ *ndh* |
| **mc^2^5872** | mc^2^6230 | Δ*RD1* Δ*panCD* Δ*ndhA* |
| **mc^2^5874** | mc^2^6230 | Δ*RD1* Δ*panCD* Δ*ndhA::*P_hsp60_ *ndhA* |

### Table S2. List of differentially expressed genes in *sdh* mutants during aero- and anaerobiosis.

| **Genes upregulated >2-fold** | | |
| --- | --- | --- |
| **Condition** | **Gene symbol/name** | **Protein function** |
| **aerobic *wt* vs Δ*sdh1*** | | |
|  | Rv0318c |  |
|  | Rv1387/PPE | PPE-family protein |
|  | Rv0867c | probable exported protein |
|  | Rv1778c | hypothetical protein |
|  | Rv1813c | conserved hypothetical protein |
|  | Rv1388/ *mIHF* | integration host factor |
| **aerobic *wt* vs Δ*sdh2*** *–* none significant | | |
| **12d hypoxia *wt* vs Δ*sdh1*** | | |
|  | Rv2612c/*pgsA* | CDP-diacylglycerol-glycerol-3-phosphate |
|  | Rv2886c | resolvase |
|  | Rv0534c/ *menA* | 4-dihydroxy-2-naphthoate octaprenyltransferase |
|  | Rv0837c | hypothetical protein |
|  | Rv1744c | hypothetical protein |
|  | Rv1755c/ *plcD* | partial CDS for phospholipase C |
|  |  |  |
| **12d hypoxia *wt* vs Δ*sdh2*** | | |
|  | Rv0249c | probable membrane anchor protein |
|  | Rv0248c | probable flavoprotein subunit of Rv0247c |
|  | Rv3492c | conserved hypothetical protein |
|  | Rv0247c | probable iron-sulphur protein |
|  | Rv3579c | putative methyltransferase |
|  | Rv2052c | hypothetical protein |
|  | Rv3578/ *arsB2* | probable arsenical pump |
|  | Rv2886c | resolvase |
|  | Rv2159c | hypothetical protein |
|  | Rv0804 | conserved hypothetical protein |
|  | Rv0341 | conserved hypothetical protein |
|  | Rv0290 | unknown hydrophobic protein |
|  | Rv0394c | hypothetical protein |
|  | Rv1576c | hypothetical protein |
|  | Rv2631 | conserved hypothetical protein |
|  |  |  |
| **Genes downregulated >2-fold** | | |
| **aerobic *wt* vs Δ*sdh1*** | | |
|  | Rv0219 | hypothetical protein |
|  | Rv3768 | hypothetical protein |
|  | Rv3239c | possible antibiotic efflux proteins |
|  | Rv1998c | conserved hypothetical protein |
|  | Rv2342 | hypothetical protein |
|  | Rv2989 | transcriptional regulator (IclR family) |
|  | Rv3615c | conserved hypothetical protein |
|  | Rv1285 | sulfate adenylate transferase, subunit 2 (cysD) |
|  | Rv2777c | hypothetical protein |
|  | Rv2987c/ *leuD* | 3-isopropylmalate dehydratase small subunit |
|  | Rv2820c | hypothetical protein |
|  |  |  |
| **aerobic *wt* vs Δ*sdh2*** | | |
|  | Rv2624c | conserved hypothetical protein |
|  | Rv2488c | transcriptional regulator (LuxR/UhpA family) |
|  | Rv0424c | hypothetical protein |
| **12d hypoxia *wt* vs Δ*sdh1*** | | |
|  | Rv2641 | conserved hypothetical protein |
|  | Rv3679 | possible anion transporter |
|  | Rv0249c | probable membrane anchor protein |
|  |  |  |
| **12d hypoxia *wt* vs Δ*sdh2*** | | |
|  | Rv2035 | hypothetical protein |
|  | Rv2204c | conserved hypothetical protein |
|  | Rv2913c | probable D-amino acid aminohydrolase |
|  | Rv3862c | hypothetical protein |
|  | Rv1471/ *trxB* | thioredoxin reductase |
|  | Rv1875 | conserved hypothetical protein |
|  | Rv0331 | putative dehydrogenase |
|  | Rv0792c | mercuric reductase/transcriptional regulator, fusion |
|  | Rv3223c/ *sigH* | ECF subfamily sigma subunit |
|  | Rv0767c | hypothetical protein |
|  | Rv2034 | transcriptional regulator (ArsR family) |
|  | Rv1169c | PE-family protein |
|  | Rv3252c | possible alkane-1 monooxygenase |
|  | Rv2641 | conserved hypothetical protein |
|  | Rv3229c/ *desA3* | acyl-[ACP] desaturase |
|  | Rv0991c | hypothetical protein |
|  | Rv0769 | similar to 7-alpha-hydroxysteroid dehydrogenase |
|  | Rv2699c | conserved hypothetical protein |
|  | Rv0186/ *bglS* | [beta]-glucosidase |
|  | Rv1813c | conserved hypothetical protein |
|  | Rv2104c | conserved hypothetical protein |
|  | Rv2203 | hypothetical protein |
|  | Rv1218c | probable ABC transmembrane transport protein |
|  | Rv3767c | conserved hypothetical protein |
|  | Rv0332 | hypothetical protein |
|  | Rv0140 | conserved hypothetical protein |
|  | Rv0791c | possible monooxygenasemonoxygenase |
|  | Rv1168c | PPE-family protein |
|  | Rv2642 | transcriptional regulator (ArsR family) |
|  | Rv1894c | some similarity to dioxygenases |
|  | Rv0847/ *lpqS* | lipoprotein |
|  | Rv0654 | putative dioxygenase |
|  | Rv3418c/ *groES* | 10 kD chaperone |
|  | Rv3160c | putative transcriptional regulator |

### Supporting Data

#### Annotation

*M. tuberculosis* has two operons annotated which could perform succinate oxidation, and to determine the role of each enzyme complex, we prepared strains with null deletions of each (Table S2). The operon containing *sdh1* consists of 3 ORFs with homology to known succinate dehydrogenase/fumarate reductase enzymes with *Rv0249c* apparently representing a fused membrane anchor subunit which is conserved in *Mycobacterium* and *Streptomyces* and can be classified as a Type B SQOR[10]. The operon containing *sdh2* also has homology to the same protein families but with a canonical structure in its membrane subunits (see below). *Rv0247c*, *Rv0248c*, and *Rv0249c* are in a predicted operon and encode a putative succinate dehydrogenase. *Rv0247c* has a conserved domain - Fer2_3[pfam13085], 2Fe-2S iron-sulfur cluster binding domain. *Rv0248c* has two conserved domains - NADB_Rossmann super family[cl09931], Rossmann-fold NAD(P)(+)-binding proteins; and Succ_DH_flav_C[pfam02910], This latter family contains fumarate reductases, succinate dehydrogenases and L-aspartate oxidases. *Rv0249c* has no conserved domains, but is probably a fusion of sdhCD to which it bears limited homology (T-COFFEE Score: 51 and 53).

The operon containing *sdhCDAB* (*Rv3316*, *Rv3317*, *Rv3318*, *Rv3319*) contains an annotated succinate dehydrogenase. *Rv3316* has a conserved domain - SQR_TypeA_SdhC_like[cd03501], Succinate:quinone oxidoreductase (SQR) Type A subfamily. *Rv3317* has a conserved domain -

SQR_TypeA_*SdhD*_like[cd03500], Succinate:quinone oxidoreductase (SQR) Type A subfamily. *Rv3318* has two conserved domains -

NADB_Rossmann super family[cl09931], Rossmann-fold NAD(P)(+)-binding proteins, and Succ_DH_flav_C[pfam02910]. *Rv3319* has a single conserved domain - *sdhB*[PRK05950], succinate dehydrogenase iron-sulfur subunit.

For more information, visit www.tbdb.org or see [11].

#### Complementation

We note that reinsertion of *Rv0247c-Rv0249c* overcompensated (i.e. decreased rate of killing) due to constitutive expression from HSP60 promoter; we concluded that these induction levels reflect the concentration of active enzyme in each condition. Thus, perfect complementation would require levels of expression which closely match *wt* levels, which led us to construct inducible vectors (Table S2, Fig. S8) which allowed us to titrate the expression of Sdh1 in later experiments (e.g. Figure S4). *M. tuberculosis* strains harboring deletions created by specialized transduction were complemented in two ways. First each operon was cloned into an integrative vector (pMV361) containing P_hsp60_ for unregulated high expression. Additionally, operons were cloned behind a novel version of the P_myc1_ tetracycline-inducible plasmid [12], designated pYUB1734, pYUB1735, & pYUB1736 (called P_tet2_ here). This is an integrated tetracycline-inducible vector which was modified to reduce background expression to undetectable levels in order to more closely match the observed expression pattern of the *sdh* genes (manuscript in preparation). One feature of this modified promoter is that its maximal induced expression is 10-fold lower than basal expression of P_hsp60_. The complementing plasmids reported here contain two tetracycline operator sites and are designated pYUB1753 and pYUB1754, containing the *Rv0247c-Rv0249c* operon and the *sdhCDAB* operon, respectively. Whereas *sdh2* showed good respiratory complementation when induced with 100 ng/mL anhydrotetracycline, a titration to 25 ng/mL was required for desirable complementation of *sdh1* (Figure S4A).

#### Metabolomics

We used stable isotope labeling to determine the predominant direction of carbon flux in our *sdh* mutants (Figure S2), and determined the proportion of each labeled metabolite with respect to all isotopologues for each intermediate. This method, previously reported by Watanabe *et. al*., made use of [1,4 ^13^C_2_]-labeled aspartic acid to determine that some portion of carbon in *M. tuberculosis* cells in anaerobiosis flows in a reverse direction, leading to the production of succinate [13]. When sufficient oxygen is available, the labeled compound ([1,4 ^13^C_2_] aspartate) is imported and transaminated to oxaloacetate where it enters the TCA cycle and loses both labeled carbons to CO_2_ production for each clockwise turn. However, in anaerobic conditions, an increasing proportion of the doubly labeled oxaloacetate is reduced to form malate, which retains both ^13^C-labeled carbons (M+2). This reverse flow continues, resulting in doubly-labeled succinate, which can be detected by a mass spectrometer as an M+2 isotopomer.

We performed experiments using the same experimental framework for the *sdh* mutant strains (figure S2) and verified that during log phase growth the unlabeled succinate pool was considerably higher in the ∆*sdh1* strain than in the parent (Figure S2A). The proportion of succinate bearing two ^13^C atoms was decreased to 41% with respect to the parental strain, whereas the proportion of malate containing ^13^C_2_ was increased over eight-fold, indicating a loss of fumarate reductase . Because the increased intracellular succinate could be due to compensatory activity by the glyoxylate shunt which forms glyoxylate and succinate from isocitrate [14] we examined the incorporation of label into glyoxylate; which indicated a nine-fold proportional decrease in ^13^C_1_ abundance, signifying a relatively small contribution from this pathway during aerobiosis. This was not the case in hypoxia, where the *Δsdh1* mutant actually accumulated ^13^C_1_ glyoxylate. Additionally, the decreased total malate in the ∆*sdh1* mutant was similar to the parental strain in proportion of unlabeled malate, but there was an eight-fold increase in the M+2 isotopomer. It is possible that decreased activity of Mez (the malate oxidoreductase) might be responsible for the buildup of malate and indeed, we detected a 1.7-fold decrease in M+1 pyruvate in this mutant. These isotope ratios are consistent with a loss of succinate dehydrogenase activity in this mutant. The ∆*sdh2* strain had similar total succinate levels as the parent, and there was a 1.4-fold increase in unlabeled succinate and a 1.3-fold increase in unlabeled malate, supporting intact succinate dehydrogenase activity. In hypoxia (Figure S2B), the proportion of M+2 malate was decreased in the ∆*sdh1* mutant 4.8-fold but the M+2 succinate was only slightly decreased, suggestive of intact or slightly increased Frd activity. The unlabeled succinate concentration (indicative of oxidative flux) was increased 0.9-fold, and M+0 malate was increased over two-fold in the ∆*sdh1* strain. Conversely, the ∆*sdh2* mutant continued to show a decrease in unlabeled succinate (0.5-fold) and a 1.3-fold increase in the proportion of unlabeled malate, indicative of intact aerobic Sdh activity (presumably) by *sdh1*.

It is important to note that the proportion of labeled intermediates using this method – even in hypoxia - is less than 10% of total signal for each intermediate, so we considered the labeled fraction of these metabolites adjusted for naturally occurring isotope abundance and compared the proportion of each isotopologue with respect to the proportion of the same molecule in the parental strain for each condition.

Because the increased intracellular succinate could be due to compensatory activity by the glyoxylate shunt which forms glyoxylate and succinate from isocitrate [14] we examined the incorporation of label into glyoxylate; which indicated a nine-fold proportional decrease in ^13^C_1_ abundance, signifying a relatively small contribution from this pathway during aerobiosis.

It is possible that decreased activity of Mez (the malate oxidoreductase) might be responsible for the buildup of malate and indeed, we detected a 1.7-fold decrease in M+1 pyruvate in this mutant. The unlabeled succinate concentration (indicative of oxidative flux) was increased 0.9-fold, and M+0 malate was increased over two-fold in the Δ*sdh2* strain.

Regarding the proposed functional reassignment; the metabolomic data is consistent with transcriptional data from *M. smegmatis* that shows a 2-fold increase in *sdh2* transcripts under hypoxic conditions but a 30-fold decrease of *sdh1* transcripts. *M. smegmatis* lacks *frdABCD*, hence Sdh2 is likely the dedicated fumarate reductase [15].

### References

1. Bardarov S, Bardarov Jr S, Pavelka Jr MS, Sambandamurthy V, Larsen M, et al. (2002) Specialized transduction: an efficient method for generating marked and unmarked targeted gene disruptions in Mycobacterium tuberculosis, M. bovis BCG and M. smegmatis. Microbiology 148: 3007–3017. Available: http://www.ncbi.nlm.nih.gov/pubmed/12368434.

2. Sambandamurthy VK, Wang X, Chen B, Russell RG, Derrick S, et al. (2002) A pantothenate auxotroph of Mycobacterium tuberculosis is highly attenuated and protects mice against tuberculosis. Nat Med 8: 1171–1174. Available: http://www.ncbi.nlm.nih.gov/pubmed/12219086. Accessed 4 August 2011.

3. Notredame C, Higgins DG, Heringa J (2000) T-Coffee: A novel method for fast and accurate multiple sequence alignment. J Mol Biol 302: 205–217. Available: http://www.ncbi.nlm.nih.gov/pubmed/10964570. Accessed 27 February 2013.

4. Rubin EJ, Akerley BJ, Novik VN, Lampe DJ, Husson RN, et al. (1999) In vivo transposition of mariner-based elements in enteric bacteria and mycobacteria. Proc Natl Acad Sci 96: 1645–1650. Available: http://www.pnas.org/cgi/doi/10.1073/pnas.96.4.1645. Accessed 26 July 2013.

5. Murry JP, Sassetti CM, Lane JM, Xie Z, Rubin EJ (2008) Transposon site hybridization in Mycobacterium tuberculosis. Methods Mol Biol 416: 45–59. Available: http://www.ncbi.nlm.nih.gov/pubmed/18392960.

6. Paglia G, Hrafnsdóttir S, Magnúsdóttir M, Fleming RMT, Thorlacius S, et al. (2012) Monitoring metabolites consumption and secretion in cultured cells using ultra-performance liquid chromatography quadrupole-time of flight mass spectrometry (UPLC-Q-ToF-MS). Anal Bioanal Chem 402: 1183–1198. Available: http://www.ncbi.nlm.nih.gov/pubmed/22159369. Accessed 25 November 2012.

7. Zilberstein D, Agmon V, Schuldiner S, Padan E (1984) Escherichia coli intracellular pH, membrane potential, and cell growth. J Bacteriol 158: 246–252. Available: http://www.pubmedcentral.nih.gov/articlerender.fcgi?artid=215405&tool=pmcentrez&rendertype=abstract.

8. Bekker M, Kramer G, Hartog AF, Wagner MJ, de Koster CG, et al. (2007) Changes in the redox state and composition of the quinone pool of Escherichia coli during aerobic batch-culture growth. Microbiology 153: 1974–1980. Available: http://mic.sgmjournals.org/content/153/6/1974.full. Accessed 16 October 2012.

9. Larsen MH, Biermann K, Tandberg S, Hsu T, Jacobs WR (2007) Genetic Manipulation of Mycobacterium tuberculosis. Curr Protoc Microbiol Chapter 10: Unit 10A.2. Available: http://www.ncbi.nlm.nih.gov/pubmed/18770603. Accessed 11 April 2013.

10. Kröger A, Biel S, Simon J, Gross R, Unden G, et al. (2002) Fumarate respiration of Wolinella succinogenes: enzymology, energetics and coupling mechanism. Biochim Biophys Acta 1553: 23–38. Available: http://www.ncbi.nlm.nih.gov/pubmed/11803015.

11. Juhnke HD, Hiltscher H, Nasiri HR, Schwalbe H, Lancaster CRD (2009) Production, characterization and determination of the real catalytic properties of the putative “succinate dehydrogenase” from Wolinella succinogenes. Mol Microbiol 71: 1088–1101. Available: http://www.pubmedcentral.nih.gov/articlerender.fcgi?artid=2680327&tool=pmcentrez&rendertype=abstract. Accessed 28 January 2013.

12. Ehrt S, Guo X V, Hickey CM, Ryou M, Monteleone M, et al. (2005) Controlling gene expression in mycobacteria with anhydrotetracycline and Tet repressor. Nucleic Acids Res 33: e21. Available: http://www.pubmedcentral.nih.gov/articlerender.fcgi?artid=548372&tool=pmcentrez&rendertype=abstract. Accessed 29 November 2012.

13. Watanabe S, Zimmermann M, Goodwin MB, Sauer U, Barry CE, et al. (2011) Fumarate reductase activity maintains an energized membrane in anaerobic Mycobacterium tuberculosis. PLoS Pathog 7: e1002287. Available: http://dx.plos.org/10.1371/journal.ppat.1002287. Accessed 3 June 2014.

14. McKinney JD, Höner zu Bentrup K, Muñoz-Elías EJ, Miczak a, Chen B, et al. (2000) Persistence of Mycobacterium tuberculosis in macrophages and mice requires the glyoxylate shunt enzyme isocitrate lyase. Nature 406: 735–738. Available: http://www.ncbi.nlm.nih.gov/pubmed/10963599.

15. Berney M, Cook GM (2010) Unique flexibility in energy metabolism allows mycobacteria to combat starvation and hypoxia. PLoS One 5: e8614. Available: http://www.pubmedcentral.nih.gov/articlerender.fcgi?artid=2799521&tool=pmcentrez&rendertype=abstract. Accessed 22 June 2011.
